# Identification and characterization of novel CD274 (PD‐L1) regulating microRNAs and their functional relevance in melanoma

**DOI:** 10.1002/ctm2.934

**Published:** 2022-07-08

**Authors:** Christoforos K. Vaxevanis, Michael Friedrich, Sandy Uta Tretbar, Diana Handke, Yuan Wang, Juliane Blümke, Reinhard Dummer, Chiara Massa, Barbara Seliger

**Affiliations:** ^1^ Institute of Medical Immunology Martin Luther University Halle‐Wittenberg Halle Germany; ^2^ Clinic of Dermatology Universitäts‐Spital Zürich Zürich Switzerland

**Keywords:** biomarker, melanoma, microRNAs, PD‐L1/CD274, therapeutic target

## Abstract

**Background:**

Immune checkpoint inhibitors directed against programmed cell death 1 (PDCD1/PD1) receptor and programmed cell death‐ligand 1 (CD274/PD‐L1) have been recently successfully implemented for the treatment of many cancers, but the response rate of tumour patients is still limited due to intrinsic and acquired resistances. However, the underlying molecular mechanisms of this limited response have still to be defined in detail. The aim of this study is to uncover processes inhibiting PDCD1/CD274 expression thereby enhancing anti‐tumour immune responses. The identification and characterization of microRNAs (miRNAs) targeting the 3′‐untranslated region (3′‐UTR) as well as the coding sequence (CDS) of *CD274* will provide the basis for a new drug development.

**Methods:**

Human melanoma cell lines and tissue samples were subjected to mRNA and/or protein expression analysis using qPCR, Western blot, flow cytometry, and/or immunohistochemistry. The data were correlated to clinical parameters. MiRNA trapping by RNA in vitro affinity purification (miTRAP) technology in combination with small RNA sequencing and different bioinformatics tools were employed to identify CD274‐regulating miRNAs.

**Results:**

Screening based on miTRAP in combination with RNAseq identified a large number of novel CD274‐regulating candidate miRNAs, from which eight selected miRNAs were functionally validated. Five out of eight miRNAs were able to significantly reduce CD274 surface expression indicating that these miRNAs directly bind to the 3′‐UTR or CDS of the *CD274* gene. The miRNA‐mediated inhibition of CD274 expression was accompanied by an increased T cell recognition. Furthermore, an inverse expression of three CD274‐regulating miRNAs and CD274 was demonstrated in melanoma lesions. A CD274 miRNA score was generated, which was associated with disease progression and reduced survival of melanoma patients.

**Conclusions:**

These data revealed a novel mechanism that miRNAs targeting the CDS of immune checkpoint genes are functional, have prognostic relevance, and also the potential for the development of novel miRNA‐based therapies.

## Graphical abstract

- Unique miRNAs targeting the 3'UTR and CDS of CD274 (PD‐L1) were identified.
- MiRNA targets successfully downregulated CD274 and affected cell‐mediated cytotoxicity.
- The created ComPDM score utilizing the expression of CD274 ‐targeting miRNAs, CD274 expression and CD8+ T cell infiltration was proven to be an independent prognostic marker for PFS of untreated melanoma patients.

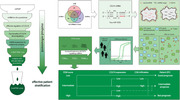

## 1 INTRODUCTION

Cluster of differentiation 274 (CD274), most commonly referred to as programmed cell death‐ligand (PD‐L1 or B7‐H1), is a type 1 transmembrane protein, which interacts with the T cell inhibitory receptor named programmed cell death protein 1 (PDCD1).^1^ The interaction of PDCD1/CD274 (PD‐L1) on the cell surface promotes T cell tolerance, minimizes autoimmune‐mediated inflammation, but also mediates the immune escape of tumour cells.^2^ CD274 is a checkpoint ligand, which is expressed by various immune cells and antigen presenting cells, but also overexpressed in different cancer cells.^3^ The latter results in a reduced elimination of tumour cells by CD8^+^ cytotoxic T lymphocytes (CTL) thereby promoting tumour progression. Indeed, higher levels of CD274 expression have been associated with disease progression, high risk of cancer mortality as well as a poor patients’ outcome.^4^ Targeted therapies blocking the CD274 (PD‐L1)/PDCD1 (PD1) interaction are a promising therapeutic option for various cancer types and already approved for the treatment of, for example, melanoma, non‐small cell lung cancer (NSCLC), renal cell carcinoma (RCC), bladder cancer, and head and neck squamous cell carcinoma (HNSCC).^5^

Despite the promising results, the objective response rate of patients with various cancer types are still low. This might be due to the high individual variation of CD274 expression mediated by cellular and soluble components of the tumour microenvironment (TME).^6^ Furthermore, it has been demonstrated that poor responders to immune checkpoint inhibitor (CPI) therapy express low CD274 levels and have a reduced T cell infiltration.^7^ Thus, there is an urgent need to understand how CD274 levels on intra‐tumoural compartments influence tumour growth and predict treatment response.

Recently, a complex regulation of CD274 expression was reported including genomic alterations, transcriptional and epigenetic control, mRNA and protein stability, post‐transcriptional regulation as well as inflammatory and oncogenic signalling.^8^ So far, various microRNAs (miRNAs), which directly bind to the 3′‐UTR of the *CD274* gene or influence the expression of other CD274 regulators, have been identified to post‐transcriptionally control the CD274 expression,^9^, ^10^, ^11^, ^12^, ^13^ while no miRNAs were reported, which bind to the coding sequence (CDS) of the *CD274* gene. Some of these CD274‐specific miRNAs identified might serve as prognostic factors and/or have a role in the resistance development to cancer immunotherapy.^14^, ^15^, ^16^ It has been suggested that miRNAs targeting CD274 might mimic the therapeutic effect of checkpoint blockade.^17^ Here, we identify a number of CD274‐regulating miRNAs interacting with the 3′‐UTR or for the first time binding to the CDS of the *CD274* gene. These miRNAs suppress the expression and function of CD274 upon transfection in melanoma cell lines and are of prognostic value for melanoma patients.

## 2 MATERIALS AND METHODS

### 2.1 Cell lines and tissue culture

The human melanoma cell lines IRNE and mel1359 used were kindly provided by G. Pawelec (University of Tuebingen, Germany) and have recently been published.^18^ These cell lines were maintained in Roswell Park Memorial Institute 1640 Medium (RPMI, Invitrogen) supplemented with 10% (v/v) foetal bovine serum (FCS) (PAA, Pashing, Austria), .1 mM non‐essential amino acids (Gibco, Dublin, Ireland), 2 mM L‐glutamine (Lonza, Basel, Switzerland), and 1% penicillin/streptomycin (v/v; PAA) at 37°C, 5% CO_2_. The HEK293T cell line was acquired from DSMZ and was cultured in DMEM medium (Sigma‐Aldrich, Munich, Germany) supplemented with 10% (v/v) FCS (PAA), .1 mM non‐essential amino acids (Gibco), 2 mM L‐glutamine (Lonza) and 1% penicillin/streptomycin (v/v; PAA) at 37°C, 5% CO_2_.

### 2.2 Human melanoma tissue samples

Tissue samples from cutaneous malignant melanoma (*n* = 46) were collected between 2008 and 2016 in the Department of Dermatology of the University Hospital of Zurich, Zurich, Switzerland and the University Hospital in Salzburg, Austria. The study was performed according to the Declaration of Helsinki and approved by the ethical committees of the University Hospital in Zurich (KEK‐ZH‐No. 647 and 800) as well as of the University Hospital in Salzburg (E‐No. 2142). The clinical data from the melanoma patients according to the American Joint Committee on Cancer (AJCC) as well as the CD274 (PD‐L1) expression and immune cell infiltration of the tumour lesions are available.

### 2.3 Transfection and luciferase (luc) reporter gene assay

For miRNA transfections, melanoma cells were seeded at 1×10^5^ cells/well in 6‐well plates and were transfected with miRNA mimics or controls (Sigma, Kawasaki, Japan), respectively, at a final amount of 10 pM using Lipofectamine RNAi MAX (Invitrogen, Carlsbad, CA, USA) according to the manufacturers’ reverse transfection protocol. Cells were harvested 48 h after transfection and directly subjected to analysis.

For the luciferase (luc) reporter assay, 1 × 10^4^ HEK293T cells/well were seeded in 96‐well plates. After 16 h, 10 ng of the reporter plasmid (pmirGLO Dual‐Luciferase miRNA Target Expression Vector, Promega, Madison, Wisconsin, USA) in combination with the respective miRNA mimic or control (Sigma) at a final concentration of 25 nM were transfected using Lipofectamine 2000 (Invitrogen) according to the manufacturers’ protocol. Luc activity was determined 48 h after transfection employing the Dual‐Glo® Luciferase substrate (Promega) according to the manufacturers’ protocol. Luminescence was determined with a Tecan Infinite 200 Pro plate fluorescence reader device. Relative luc activity was determined by normalizing the firefly reporter to Renilla luc activity as internal reference. Mutated plasmids carrying deletions of the in silico predicted binding sites were generated using the Q5 directed mutagenesis kit (NEB) to determine specific binding of the miRNAs identified on the different parts of CD274. The deleted regions were 100–800 nt long so that the binding sites of each miRNA of interest are deleted at the same time. The primer pairs for the mutated plasmids were designed using NEBaseChanger. No plasmid was created for the binding site of miR‐155 as these results have been already published.^14^

### 2.4 RNA isolation, cDNA synthesis, and quantitative PCR

Total RNA was isolated from cultured cells using the NucleoSpin® miRNA kit (Macherey & Nagel, Dueren, Germany) according to the manufacturers’ instructions followed by DNase I treatment (NEB, Ipswich, USA). RNA quality and quantity were assessed by spectrophotometric analysis and 500 ng of total RNA was used for cDNA synthesis (RevertAid H Minus First Strand cDNA synthesis kit, Fermentas, Waltham, MS, USA).

For miRNA‐specific cDNA synthesis, a miRNA‐specific stem‐loop primer was used, while reverse transcription of mRNAs required a random hexamer primer (Fermentas). Expression levels were analysed by qRT‐PCR using GoTaq® qPCR Master Mix (Promega). For all primer pairs, an annealing temperature of 60°C was used. Relative changes of mRNA/miRNA amounts were determined by the ΔΔC_t_ method using U6 snRNA, U44 snoRNA, U46 snoRNA, and U47snoRNA for normalization. All primers are listed in Table S1. For miRNA analysis of paraffin‐embedded tissues, the recently published protocol was used.^18^

### 2.5 Flow cytometry

For flow cytometric analyses the following monoclonal antibodies (mAbs) were employed: APC conjugated CD274 (PD‐L1) (clone MIH3, Invitrogen) and FITC conjugated annexin V (Miltenyi Biotech, Bergisch‐Gladbach, Germany). Cells were stained with the annexin V mAb as recommended by the manufacturers and then stained with the anti‐PD‐L1 mAb at a 1:100 dilution for 30 min at 4°C. The measurements were performed on a BD LSR Fortessa unit (Becton Dickinson, Heidelberg, Germany). FlowJo software (FlowJo, LLC) was used for analyses and to display flow cytometry results. The results are presented in histograms as mean fluorescence intensity (MFI).

### 2.6 Western blot analysis

For the quantification of CD274 (PD‐L1) protein expression, total protein from transfected and control cells was extracted and quantified using the Pierce BCA protein assay kit (Thermo Fisher, Waltham, MS, USA). 50 μg of total protein was loaded on 10% denaturing polyacrylamide gels and transferred onto nitrocellulose membranes (GE Healthcare, Chicago, IL, USA). The membranes were blocked in TBS‐T, 5% (w/v) skimmed milk for 1 h and incubated with the primary antibodies directed against PD‐L1 (CAL10 clone, abcam, 1:1000 dilution in TBS‐T, 5% (w/v) bovine serum albumin and β‐actin (clone, Abcam, Cambridge, UK, 1:2000 dilution in TBS‐T, 5% (w/v) BSA) overnight at 4°C. For detection, a secondary antibody conjugated with horseradish peroxidase (HRP) and the Lumi‐Light substrate (Roche Applied Science, Basel, Switzerland) were applied. The signal was recorded with a LAS3000 camera system (Fuji LAS3000, Fuji GmbH, Düsseldorf) using the Image Reader LAS3000 software. The immunostaining signals were subsequently analysed using the ImageJ software (NIH, Bethesda, MD, USA). Quantification was done by setting the peak values of the control protein (β‐actin) for each loaded sample as “1”.

### 2.7 miRNA enrichment assay (miTRAP) and small RNA sequencing

To identify potential miRNAs targeting the 3′‐UTR or the CDS of the *CD274 (PD‐L1)* gene, the miTRAP method was employed, as recently described.^19^ The miTRAP eluates were either used for cDNA synthesis of candidate or control miRNAs or subjected to small RNA sequencing (RNA‐seq), which was carried out at Novogene (Hong Kong, China) and analysed as published.^19^ A selected summary of the miRNA read counts obtained for the *CD274* (PD‐L1) CDS and MS2 control along with the sequences are provided in Table S2, while the full sequencing reads are shown in Table S3.

### 2.8 Stimulation of peripheral blood mononuclear cells and cytotoxicity assays

In order to investigate the functional potential of the miRNA‐mediated CD274 (PD‐L1) regulation, cytotoxic assays were performed. Briefly, total peripheral blood mononuclear cells (PBMCs) obtained from healthy HLA‐A*03 donors were isolated using density gradient centrifugation (Biocoll, Biochrom GmbH, Berlin, Germany) and cultured in X‐VIVO15™ medium. Antigen‐specific cytotoxic T cells were generated using the following protocol. PBMC were seeded in 6‐well plate at a density of 200 000 cells/well. After 24 h, non‐adherent lymphocytes were transferred into new wells and cultured in the presence of 200 U/mL human recombinant IL‐2 (Proleukin, Clinigen, Burton upon Trent, UK) whereas adherent monocytes were supplemented for 5 days with 100 ng/mL rh‐GM‐CSF (Sanofi, Paris, France) and 100 ng/mL rh‐IL‐4 (Immunotools, Friesoythe, Germany) to induce dendritic cell differentiation. After additional 24 h culture in the presence a lysate from 2 × 10^5^ mel1359 cells obtained with heat shock (3 × 2′ cycles between liquid nitrogen and 37°C water bath), they were used to stimulate the non‐adherent lymphocytes in the presence of small concentration of IL‐2 (20 U/mL). After one week, an additional cycle of stimulation with tumour lysate pulsed cells was performed.

Due to the low basal expression on CTL obtained with both protocols, PDCD1 (PD1) expression was induced 24 h prior to the assay by anti‐CD3 (plate coated, 1 h 37°C, eBioscience, San Diego, CA, USA) and anti‐CD28 (BD Pharmigen) stimulation in the presence of 20 ng/mL IL‐6 (kindly provided by S. Rose‐John, Institute of Biochemistry, University of Kiel, Kiel, Germany), 20 ng/mL IL‐12 (Immunotools) and 50 ng/mL TGF‐β1 (Biolegend, San Diego, California, USA).

Mel1359 cells were transfected as described above and stained with CellTrace™ CFSE (Invitrogen) for the mock control and the Cell Proliferation Dye eFluor™ 670 (eBioscience) for the target miRNAs (miR‐17, miR‐155) according to manufacturer's protocol. The mock‐transfected, FITC‐labelled and the miRNA‐transfected, APC‐labelled mel1359 cells were mixed at a 1:1 ratio and further incubated with effector cells for 4 h at an effector to target (E:T) ratio of 5:1 and 10:1, respectively. The effector cells primed with the mel1359 lysate were used to assess cytotoxicity (primed PBMCs), while the same PBMCs without priming (unprimed PBMCs) with the mel1359 lysate were used as a control. Before acquisition of the LSR Fortessa (BD Becton Dickinson), the cells were stained with Zombie aqua (Biolegend) for viability assessment. Cytotoxicity was assessed based on the difference in percentages of the mock‐transfected, FITC‐positive and miRNA‐transfected, APC‐positive live mel1359 cells after co‐culture with primed PBMCs compared to the unprimed PBMCs with lack of specificity for mel1359. The experimental data were validated by co‐culture of primed PBMCs with the wild type FITC‐labelled mel1359 and APC‐labelled mel1359 cells in the absence or presence of anti‐PD‐L1 antibody (clone MIH1, Invitrogen) for 1 h at 37°C.

### 2.9 Statistical analysis

Mann‐Whitney tests, *t*‐tests, chi‐square tests, and survival analysis were performed and plotted using GraphPad Prism v.8.0.1. Multivariate Cox regression survival analyses were performed using the IBM SPSS statistics 25. The receiver operating characteristics (ROC) area under the curve (AUC) was used to plot the PDM score, CD8^+^ T cell infiltration to assess the predictive accuracy of CD274 expression. The data were considered significant with a *p* value < .05. Statistical significance is indicated with one star for *p* < .05, two stars for *p* < .001 and 3 stars for *p* < .001.

## 3 RESULTS

### 3.1 Identification of CD274 (PD‐L1) targeting miRNA candidates

**FIGURE 1:**
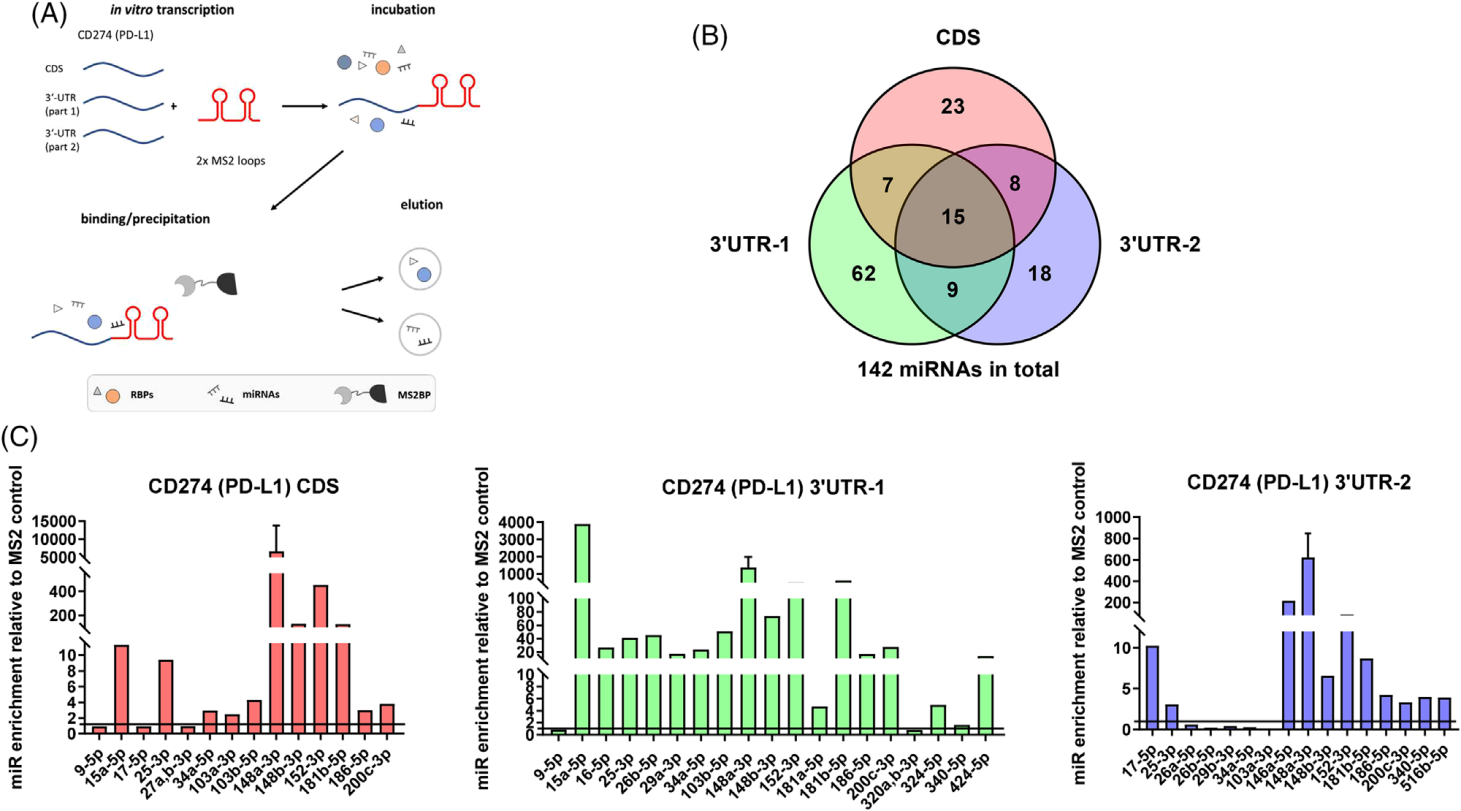
Identification of PD‐L1 targeting miRNAs. (A) Graphical representation of the miTRAP method followed for the identification of PD‐L1 specific miRNAs. (B) Venn diagram of the miRNAs binding to the three different parts of PD‐L1. (C) Validation of selected candidates via qPCR. The relative expression of the target miRNAs is depicted in comparison to the enrichment found in the MS2 control, which is set to 1 (black line)

So far, the post‐transcriptional regulation of CD274 is poorly understood in detail. Although some miRNAs targeting CD274 have been already published,^11^, ^20^, ^21^ the total number of identified miRNAs appears to be rather low, in particular considering the large size of the PD‐L1 3′‐UTR (2691 nt) and the possibility of immune modulatory miRNAs binding to the CDS. Therefore, we aimed to identify novel PD‐L1 regulating miRNAs using the miTRAP method (Figure 1A) followed by RNA‐seq.^22^, ^23^ As a bait, in vitro transcribed RNA representing the CDS or the 3′‐UTR sequence, which was split due to its large size into two parts (part 1: 1–1419 nt; part 2: 1288–2691 nt), were used. Since an important requirement for the outcome of the miTRAP analysis is the selection of a suitable cell line, the CD274 mRNA and protein expression profiles of 49 different melanoma cell lines were monitored regarding a discordant CD274 expression as indicator of a post‐transcriptional regulation. Based on these data the melanoma cell line IRNE was chosen for miTRAP analysis, since it showed a low surface expression of CD274 despite high mRNA levels (data not shown), indicative of a posttranscriptional regulation of *CD274*. After miRNA enrichment at the respective *CD274* in vitro transcript, co‐purified miRNAs were assessed by high‐throughput small RNA‐seq. Libraries were generated from the *CD274* CDS, both parts of the 3′‐UTR and MS2 control miTRAP eluates. The RNA‐seq results revealed high levels of co‐precipitated miRNAs within the eluates of the different regions (Figure 1B) with 142 unique miRNAs detected. The high reliability of this method was underscored by the fact that more than 75% of the already known CD274 specific miRNAs were identified in the eluate using this approach.^24^ Fifteen/142 miRNAs were found common in the eluates of the CD274 CDS and both 3′ UTRs. The highest number of miRNAs identified (*n* = 62) were found the eluates of the 3′ UTR‐1 as bait. After evaluation of the normalized read counts (TPM) and setting up stringent criteria for the identification of potential CD274‐regulating miRNA candidates, such as (i) high normalized read counts and enrichment ratio, (ii) binding sites in at least two of the investigated *CD274* sequences, (iii) multiple binding sites and (iv) binding prediction by at least three out of four applied tools (miRDB, miRWalk, TargetScan, RNA22),^25^, ^26^, ^27^, ^28^ 23 miRNA candidates potentially regulating PD‐L1 were selected, from which 11 miRNAs were candidates binding to the CDS. Their expression was validated by qPCR analysis of the miTRAP eluates as presented in Figure 1C. From the confirmed miRNAs, a panel of in total six novel miRNA candidates targeting the 3′‐UTR (*n* = 4) or CDS (*n* = 2) of PD‐L1, namely miR‐26a/b‐5p, miR‐29a‐3p, miR‐103b, miR‐181b‐5p, and miR‐186‐5p, were chosen to further investigate their contribution in CD274 regulation based on expression levels, number of predicted binding sites and immune modulatory functions in the literature. Based on its direct binding on CD274 as well as the regulation of c‐fos, a known transcription factor of CD274, miR‐155‐5p^14^, ^29^ was included in the analysis despite no detection in the miTRAP eluates (low/no expression in the chosen IRNE cell line). In addition, miR‐17‐5p was added as a positive control in the assays.^30^

### 3.2 Regulatory effect of selected miRNAs on the CD274 protein level

**FIGURE 2:**
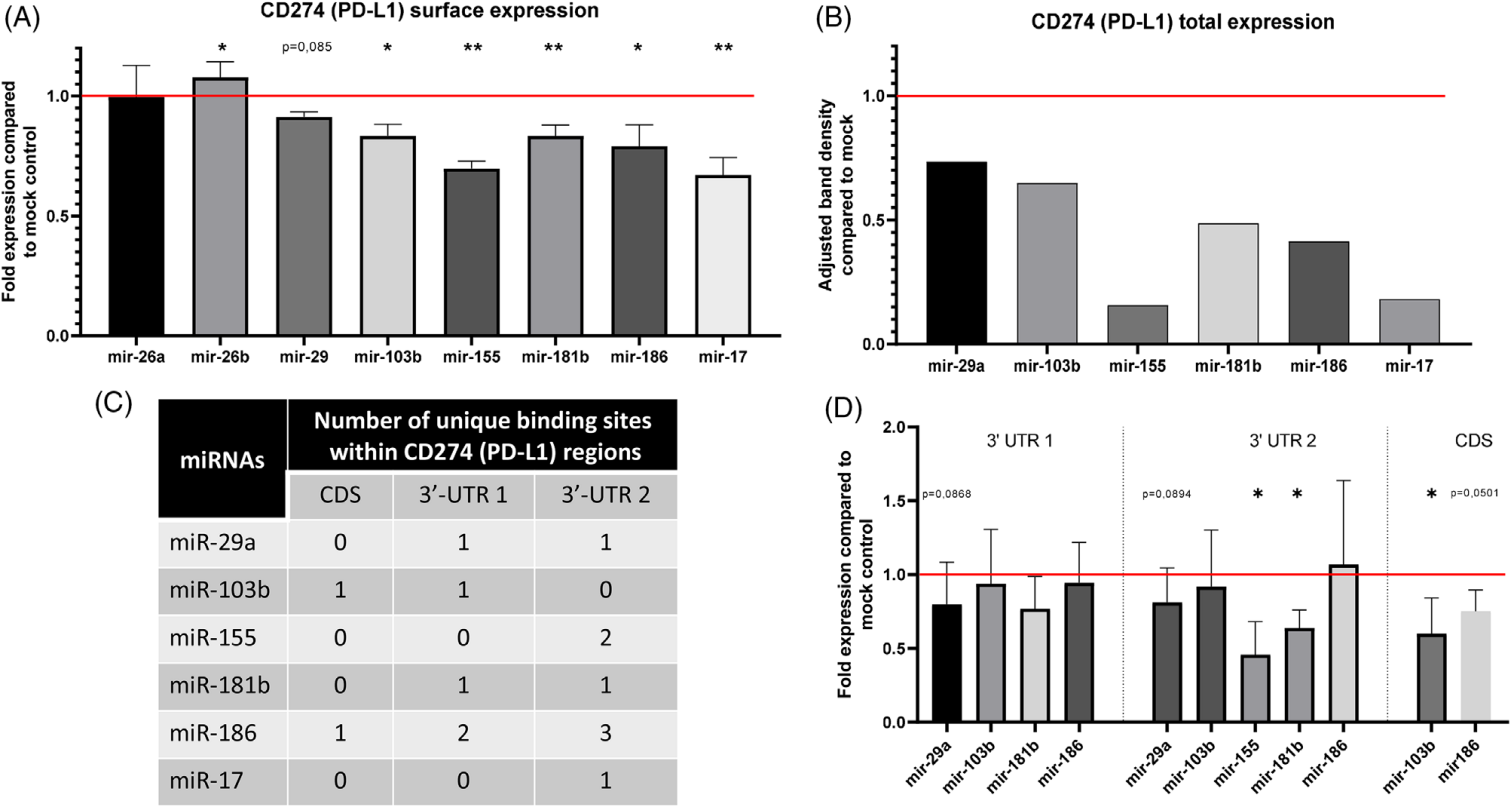
Modulation of CD274 (PD‐L1) expression by overexpression of selected miRNA mimics and determination of binding to the 3′‐UTR or CDS. The relative surface expression (A) and total expression (B) of CD274 (PD‐L1) after transfection of mel1359 with the selected miRNAs was determined by flow cytometry and Western blot analysis, respectively. Shown are the mean −/+ SE/SD of the x‐fold change to the mock control (red line) from four different experiments. Paired *t*‐tests were conducted using the MFI values of CD274 obtained in each experiment and not the depicted ratios compared to the mock control. (C) Number of putative binding sites of the various miRNAs on the three different parts of the *CD274* gene as predicted by Target Scan. (D) Relative luminescence levels after transfection with the candidate miRNAs compared to the mock control (red line) using dual luciferase assay. MiRNAs were only tested on the parts predicted to have a binding site

In order to determine the possible regulatory effects of the identified miRNAs on CD274 expression in vitro, melanoma cells were transfected with respective selected miRNA mimics. The cell line mel1359 was chosen due to its high transfection efficiency and moderate to high levels of both mRNA and protein CD274 expression (data not shown) to minimize possible pre‐existing post‐transcriptional regulation. A significantly downregulated CD274 surface expression was found upon transfection of mimics for 5/8 candidates (miR‐103b, miR‐155‐5p, miR‐181b‐5p, miR‐186‐5p, and miR‐17‐5p), which ranged between 10% to 30% (Figure 2A), while miR‐29a showed also a decrease, although not significant (p=0,085). On the other hand, the overexpression of the two candidates miR‐26a and miR‐26b either did not affect, or resulted in an upregulation of the CD274 surface expression (Figure 2A). Since these results were not within the scientific scope of our study, miR‐26a and miR‐26b were omitted for further analysis. Using flow cytometry, miR‐103b and miR‐181b transfectants displayed a reduced CD274 expression between 10% and 20%, miR‐186 overexpression downregulated CD274 expression up to 25% and the miR‐155 mimic used as control reduced CD274 surface expression to >30% (Figure 2A). These results were further confirmed by Western blot analysis of the transfectants demonstrating a reduced expression of CD274 (Figure 2B).

### 3.3 Assessment of miRNA binding on selected CD274 (PD‐L1) sequences

In order to confirm whether the observed downregulation of CD274 expression upon transfection with the candidate miRNAs is a direct effect of their binding to the *CD274* 3′‐UTR or CDS, respectively, and not a subsequent result of the regulation of this pathway (e.g., c‐fos regulation by miR‐155 and miR‐181b), dual luciferase (luc) assays were conducted. The assays were carried out for the miRNAs with in silico predicted binding sites and their respective *CD274* regions (Figure 2C). As shown in Figure 2D, the presence of a miRNA binding site did neither guarantee a decrease in the luc activity nor did the existence of multiple binding sites result in an increased reduction of luc activity (Figure 2D). The strongest effects were found with the miRNAs affecting the second part of the 3′‐UTR (3′‐UTR‐2) with miR‐155 reducing the activity >50% compared to the mock control. Interestingly, miR‐181b was the only selected miRNA with a signal reduction in both 3′‐UTR segments, although the highest effects were found on the second part of the 3′‐UTR. The second highest decrease in luc activity of 50% was for miR‐103b‐5p targeting the CDS, while the CDS targeting miR‐186‐5p resulted in an approximately 20% downregulation of luc activity. The specific binding of these miRNAs to the predicted binding sites was demonstrated by luc assays using plasmids with deleted binding sites. As shown in Figure S1, most of the miRNAs showed no inhibition of the luc activity when the predicted binding sites were deleted (Figure S1).

### 3.4 Function of the CD274 (PD‐L1)‐mediated downregulation

**FIGURE 3:**
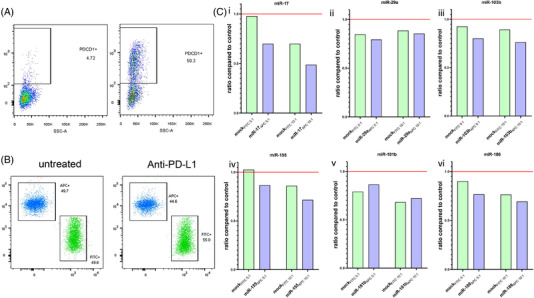
Effect of CD274 (PD‐L1) candidate miRNAs on T cell responses. (A) PDCD1 (PD1) expression on CD8^+^ T cells left untreated (i) or stimulated for 24 h with the cytokine cocktail after the activation protocol (ii) two representative plots from PBMC stimulated with the DC/IL2 out of three different experiments. (B) Dot plots of the gated mel1359 cells with (left) and without (right) prior treatment of wild type APC‐labelled cells with an anti‐PD‐L1 blocking antibody. In the presented dot plots, negative cells (unstained PBMCs) as well as double positive cells (FITC‐/APC‐labelled doublets) were excluded using a quadrant gate. (C) Mixture of FITC‐labelled mock control (light green) and APC‐labelled miRNA‐transfected (light blue) mel1359 were co‐cultured with antigen specific stimulated PBMC. After 4 h co‐culture the amount of live tumour cells was evaluated upon live aqua staining. Shown are the ratio of live mock, or miRNA transfected at the 5:1 and 10:1 E:T ratio versus the control (i–vi) (unprimed effector cells) (*n* = 1)

After the mechanistic relationship between our candidate miRNAs and PD‐L1 expression was established, it was investigated whether the miRNA‐induced PD‐L1 downregulation could enhance tumour susceptibility to CTL. Due to the low basal PDCD1 (PD1) surface expression on the T cells at the end of the activation protocols (Figure 3Ai), cells were further treated with a cocktail of stimulants (anti‐CD3/anti‐CD28, IL‐6, IL‐12, TGF‐ β1) that induced PDCD1 expression in almost half of the CTL (Figure 3Aii). After co‐culture with primed PBMCs, FITC‐labelled and APC‐labelled mel1359 cells had similar viability (Figure 3B). Validation of the PD‐L1‐related cytotoxicity was achieved by adding a blocking anti‐PD‐L1 antibody to the APC‐labelled mel1359 cells. Anti‐PD‐L1 antibody treatment resulted in a decreased survival of APC‐labelled cells when compared to untreated FITC‐labelled mel1359. In our miRNA experiments, in most cases an effector: tumour (E:T) ratio of 5:1 showed the most prominent differences between FITC‐labelled, mock transfectants, and APC‐labelled cells transfected with the miRNAs of interest. In general, miR‐17, miR‐155, and miR‐186 leading to a stronger downregulation of CD274 showed the highest differences between miRNA and mock transfectants in the cytotoxic assay (Figure 3Ci,iv,vi). Using primed PBMCs, the miR‐17_APC_ cells showed an approximately 30% and 50% reduction compared to the 3% and 30% reduction of the FITC‐labelled mock‐transfected cells at an E:T ratio of 5:1 and 10:1, respectively, compared to unprimed PBMCs (Figure 3Ci). Similar results were obtained for mel1359 cells transfected with miR‐186, which were predominantly killed more at a 5:1 E:T ratio. The strongest cytotoxic effect for miR‐155 transfected cells was obtained at an E:T ratio of 10:1 with a 15% increased killing of APC‐labelled miR‐155 cells compared to the FITC‐labelled mock transfectants (72% miR‐155_APC_ alive to 86% mock_FITC_), while comparable effects were observed for miR103b transfectants at E:T ratio of 5:1 (Figure 3Ciii,vi). The low decrease of CD274 upon miR29a transfection was not sufficient to demonstrate a significant effect on T cell cytotoxicity (Figure 3Cii). Despite the reported effect of miR181b on CD274 expression, there were no apparent differences in T cell‐mediated killing between the miRNA‐transfected cells and mock controls Moreover, since only 50% of T cells express PDCD1 (PD1), an increased cytotoxicity could be observed for the FITC‐labelled mock transfectants at higher E:T ratios, but was always lower compared to the respective cytotoxicity reported for the miRNAs that showed an effect (Figure 3C).

### 3.5 Expression of CD274‐regulating miRNA candidates on melanoma samples

**FIGURE 4:**
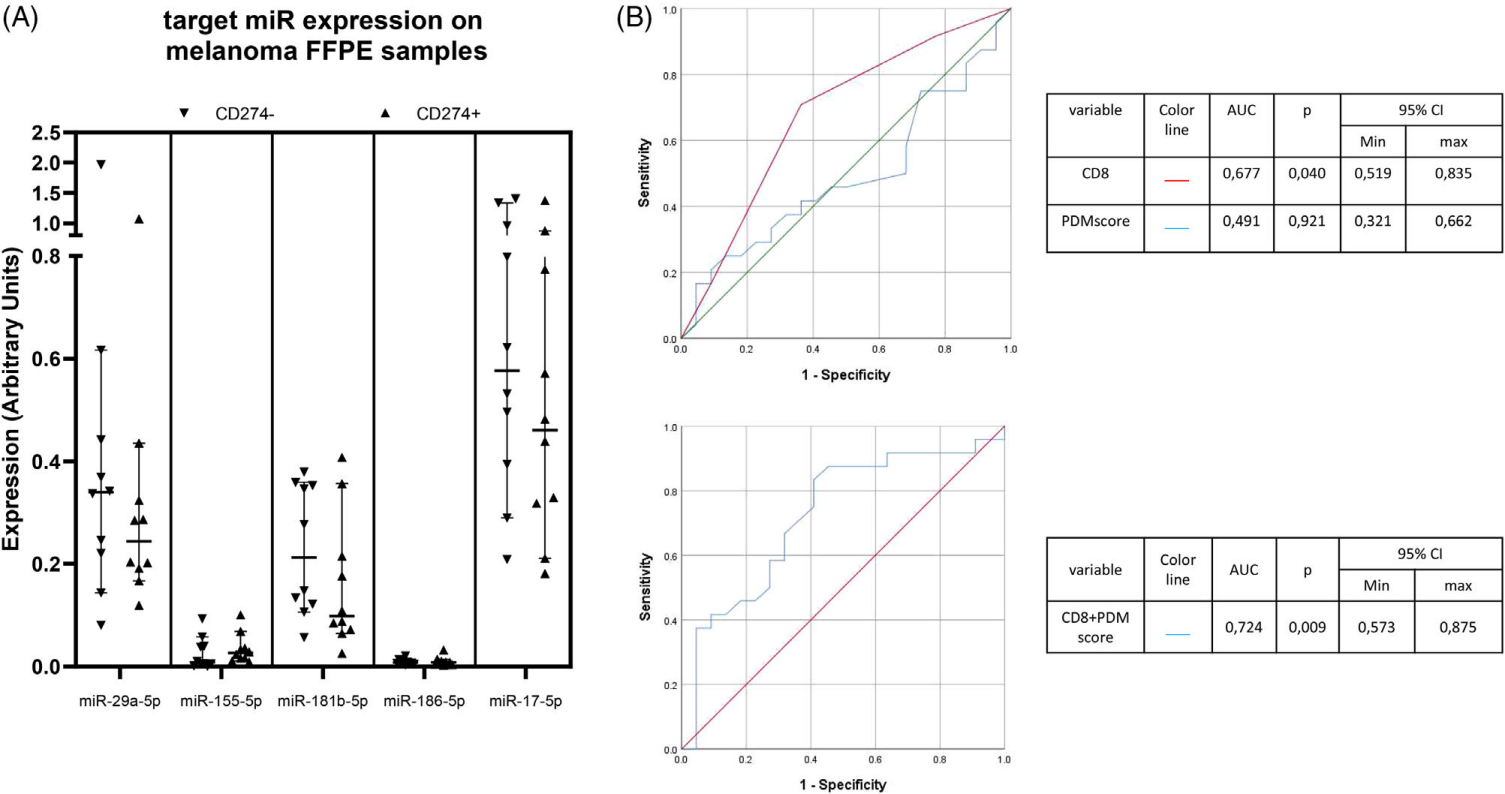
Clinical relevance of identified CD274 (PD‐L1) specific miRNAs. (A) Expression of the miRNA candidates in FFPE samples of melanoma upon their subdivision based on the PD‐L1 expression level determined by IHC. Patients negative for CD274 staining are depicted with a downwards facing triangle, while patients with a positive staining for CD274 with an upward triangle. (B) Predictive capability of CD8^+^ T cell infiltration and the PDM score generated regarding CD274 expression in the lesions of melanoma patients. The ROC curves suggest a low predictive value of CD8^+^ T cell infiltration, but none for the PDM score (top). Combination of both parameters after binomial regression revealed the importance of the PDM score leading to an increase of the AUC (bottom). The diagonal line indicates the AUC = .500 in green, while the colours of the ROC curves for each variable are shown in the respective tables along with the *p* values and 95% confidence interval

FFPE samples from 46 melanoma patients were collected at the University Hospital of Zurich, Switzerland. The clinicopathological characteristics of the patients are summarized in Table 1. Evaluation of miRNA expression highlighted that miR‐29a, miR‐181b, and miR‐17 had consistently higher expression levels in all melanoma samples in comparison to miR‐155 and miR‐186 (Figure 4A). By comparing the miRNA expression pattern with CD274 positivity, a slightly lower expression of miR‐29a and miR‐181b was detected on CD274^+^ melanoma samples, but these differences were not statistically significant. In order to determine a possible differential regulation of the miRNAs between CD274^–^ and CD274^+^ patients a possible interplay between the miRNA targets, a correlation matrix was generated (Figure S2A). Despite some correlations were found in all patients regardless of their CD274 status, CD274^+^ melanoma lesions showed a stronger correlation between miR‐186 and miR‐155/miR‐181b, while miR‐29a and miR‐17 were more strongly correlated in CD274^–^ patients. Some correlations unique to CD274^+^ lesions, for example, miR‐181b, appeared to have strong association with all investigated miRNAs (Figure S2B).

**TABLE 1:** Patient clinicopathological characteristics

**Median age**	**Range**
63	21–82
Sex	N
Male	18 (39%)
Female	28 (61%)
Tumour size (AJCC staging)	
T2	13 (28%)
T3	17 (37%)
T4	16 (35%)
N status (AJCC staging)	
N0	29 (63%)
N1	11 (24%)
N2	1 (2%)
N3	5 (11%)
Ulceration	
Not ulcerated	19 (41%)
Ulcerated	27 (59%)
CD274 (PD‐L1) status	
Negative	22 (48%)
Positive	24 (52%)

### 3.6 Generation of the CD274 (PD‐L1) miRNA (PDM) score

Since the individual analysis of the miRNA candidates was not sufficient to distinguish CD274 expression, combinatorial analyses were performed. Due to the fact that each of the five miRNA candidates analysed caused different effects on the CD274 surface expression upon transfection, their impact on the amount of reduction was used to further stratify them. MiRNAs resulting in less than a 10% reduction of CD274 expression in the mel1379 melanoma model were attributed a score of 1 (e.g., miR‐29a), while miRNAs reducing the CD274 expression level up to 20% or >20% were scored 2 (e.g., miR‐181b) or 3, respectively (Figure S3A). Moreover, individual miRNA expression levels within the melanoma patients were separated into quartiles. Low miRNA expression levels (first quartile) were considered as weak contributors and scored with 0 points (Figure S3B), while miRNAs reaching an expression level within the higher quartiles were categorized with an additional miRNA score (maximum: 3) for each subsequent quartile (Figure S3B). As a result, each patient received five individual miRNA scores (miR‐29, miR‐155, miR‐181b, miR‐186, miR‐17). The total sum of all individual miRNA scores for each patient represented the patients’ CD274 miRNA score (PDM score) with possible values spanning between 0 and 12 (Figure S3C).

### 3.7 Correlation of the PDM score with the patients’ clinicopathological characteristics

**FIGURE 5:**
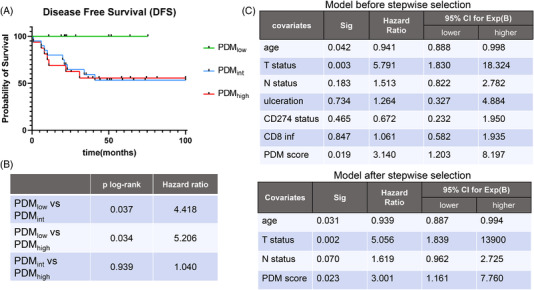
Clinical relevance of the PDM score. (A) Kaplan–Meier curves for the disease‐free survival of the melanoma patients with a low (green), intermediate (blue), and high (red) PDM score. (B) Univariate analysis of the three PDM score related groups along with the significance and hazard ratio. (C) Multivariate cox regression analysis table taking into account the major clinicopathological characteristics of the patients as well as the PDM score, before and after stepwise selection for the independent variables. Statistical significance and the hazard ratio (Exp(B)) with its 95% CI are presented

In order to further investigate the possible significance of the PDM score generated, arbitrary thresholds were set based on the miRNA expression patterns to separate patients in three distinct groups; PDM_low_ (PDM score <= 3), PDM_int_ (PDM 3 < score <= 7), and PDM_high_ (PDM score > 7). Comparisons between the three PDM groups regarding the major prognostic clinicopathological characteristics (T status, N status, ulceration) as well as the CD274 status did not reveal significant differences (Figure S4A). The subsequent correlation of the patient PDM score with the IHC data showed no direct connection with CD274 expression. In contrast, patients with a high CD8^+^ T cell infiltration had significantly higher PDM scores (Figure S4B). This relationship was further investigated after generating ROC curves for the identification of the predictive capacity of the generated PDM score. Both the CD8^+^ T cell infiltration and PDM score were analysed regarding their ability to predict CD274 expression in the melanoma patients analysed. CD8^+^ T cell infiltration showed an AUC of .677, whereas the PDM score alone was not convincing in predicting CD274 expression (Figure 4B, top). In contrast, combination of both parameters significantly increased their prediction threshold with an AUC of .724 (95% CI, .573–.875) suggesting some predictive value of the PDM score upon taking CD8^+^ T cell infiltration into account (Figure 4B, bottom). Furthermore, survival analyses demonstrated that patients with an intermediate to high PDM score had a significantly lower disease‐free survival (DFS) than patients in the PDM_low_ group (Figure 5A). In particular, patients with a PDM score > 3 (PDM_int_, PDM_high_) had 4.4–5.2 times higher risk to relapse compared to patients with a PDM score < 3, respectively (Figure 5B). However, it is noteworthy that the PDM score was not linked in this analysis to the PD‐L1 expression. Multivariate survival analysis not only demonstrated a higher significance of the PDM score groups compared to N status and ulceration (Figure 5C), but also represented an independent prognostic factor together with the T status for patients’ DFS (Figure 5C) in our limited patient cohort. It is noteworthy that the DFS of our cohort was only examined at diagnosis, after surgery and prior to immunotherapy due to the unavailability of post treatment FFPE samples for comparison of miRNA expression.

### 3.8 Prognostic value of the PDM score by combining CD274 expression and immune infiltration

**FIGURE 6:**
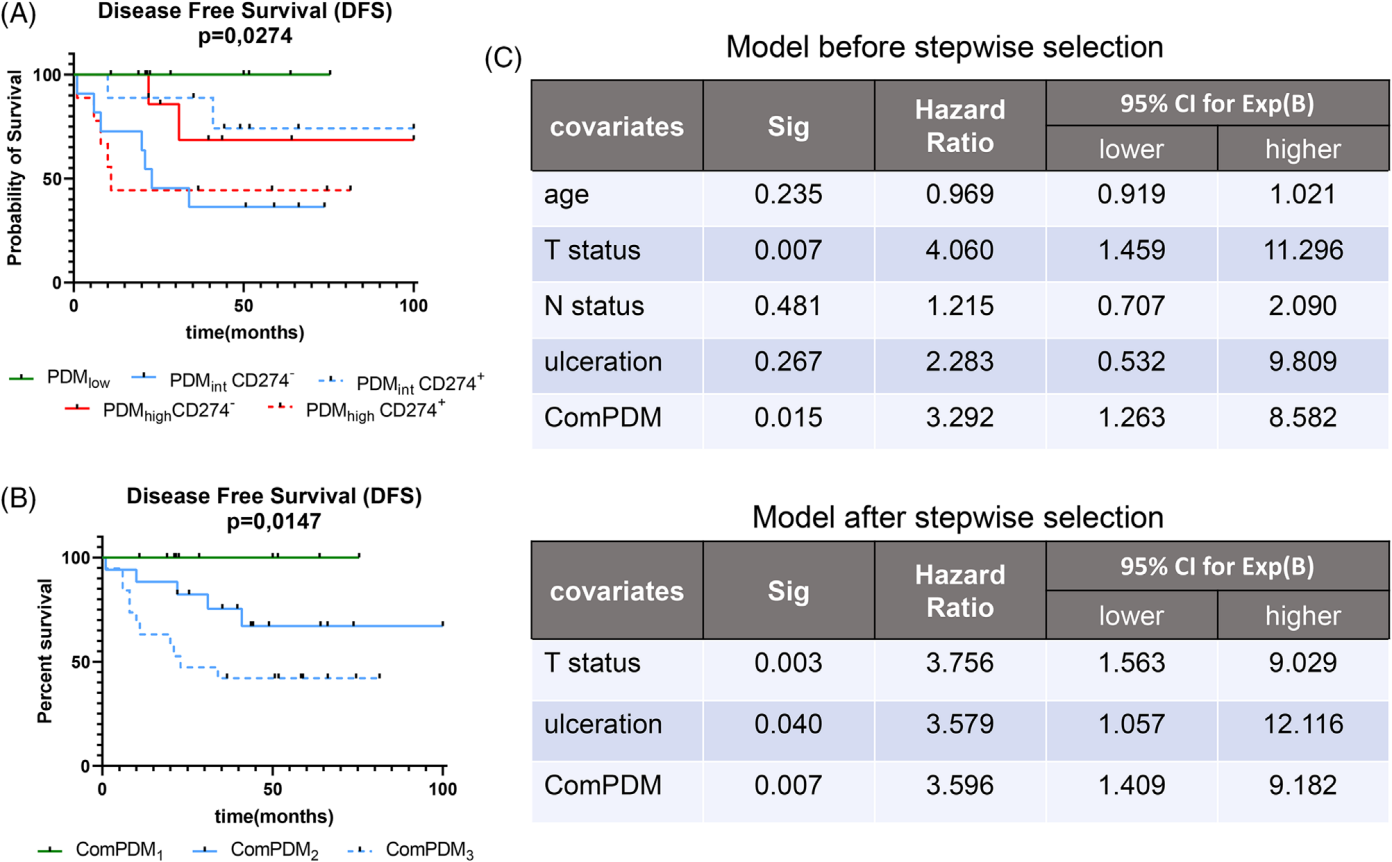
Correlation of patients’ survival by combination of the PDM score and CD274 status. (A) Kaplan–Meier curves for patients´ DFS in relation to their PDM score (low in green, intermediate in blue, high in red) and CD274status (negative with continuous lines, positive dotted lines). (B) Kaplan–Meier curves of the patients according to their comPDM status. (C) Multivariate cox regression analysis table combining the major clinicopathological characteristics of the patients with the new comPDM score, before and after stepwise selection for the independent variables. Statistical significance and the hazard ratio (Exp(B)) with its 95% CI are presented

Since the immune infiltrate might contribute to the CD274 expression as well as the miRNA profile, it was determined whether the PDM scoring system was dependent on a combination of the CD274 positivity and the frequency of T cell infiltration. As expected, patients’ DFS varied in the PDM_high_ and PDM_int_ score groups and was inversely associated with the CD274 expression. Despite a high PDM score, the CD274 positivity was accompanied by a lower DFS (Figure 6A). The opposite was found for the PDM_int_ group. In addition, the immune cell infiltration was analysed in the different PDM patients’ groups. In PDM_int_ patients, but not in the other two groups, the expression of CD274 was accompanied by a high CD8^+^ and CD4^+^ T cell infiltration, (Figure S5). Based on this information, a combined signature (comPDM) was generated that simultaneously utilized the PDM score, the CD274 positivity and CD8^+^ T cell infiltration. The comPDM_1_ group only consisted of the former PDM_low_ patients regardless of CD274 positivity and CD8^+^ T cell infiltration. The medium prognostic group, termed comPDM_2_, includes PDM_high_CD274^–^ patients as well as PDM_int_ CD8^high^ patients (Figure 6B), while the comPDM_3_ group consisted of PDM_high_CD274^+^ and PDM_int_CD8^low^ patients. Thus, this combination identified a final signature, which was able to stratify the melanoma patients into three groups with distinct prognosis (Figure 6B). Finally, multivariate analysis suggested that the comPDM signature is an independent prognostic factor with a prognostic value comparable to the T status and a hazard ratio of 3.596 between the comPDM groups (Figure 6C) in the small cohort of patients analysed.

## 4 DISCUSSION

In our present study, we were able to identify miRNAs targeting both the CDS and the 3′‐UTR of the *CD274* mRNA by using the miTRAP technology in combination with RNA sequencing of the eluate. This strategy allowed not only the identification of already known CD274‐specific miRNAs and in silico predicted candidate miRNAs, but also a large number of other novel miRNAs.^9^, ^24^, ^31^ From the various miRNAs with different enrichments in the miTRAP protocol,^19^, ^22^ only a limited number really modulated the CD274 protein expression in the reporter assay. The observed effects on CD274 expression, were neither related to the miRNA levels of enrichment in the miTRAP eluates nor to the number of their in silico predicted binding sites. These findings could be partially attributed to the constitutive expression levels of the different miRNA candidates in the mel1359 and IRNE cell lines used for miTRAP, but also suggests the possible existence of additional mechanisms involved in the miRNA‐mediated post‐transcriptional regulation.^32^ For example, long non‐coding RNA sponges could be involved, but this might still not explain the discrepancy observed on the effects of miR‐186 using different parts of the 3′‐UTR. Despite these questions, a panel of miRNAs that consistently downregulated the CD274 protein expression were identified, which were more sensitive to T cell cytotoxicity than cells treated with the anti‐PD‐L1 antibody. Our study not only validates known and novel PD‐L1‐regulating miRNAs binding to the 3′ UTR, but extends it to miRNAs, which bind to the CDS. The CDS has a limited impact on target miRNA abundance, but inhibit translation. These miRNAs increase the functional repertoire of posttranscriptional control and regulation of the protein abundance of, for example, alternative splice variants.^33^ Indeed, miRNAs targeting the CDS of the immune checkpoint HLA‐G have been recently described.^19^ Thus, miRNAs directed against both the 3′ UTR and CDS of PD‐L1 represent a promising approach for enhancing therapeutic efficacy. Finally, miRNAs targeting directly *CD274* mRNA and simultaneously regulating other factors of the CD274 pathway (e.g., miR‐155 and miR‐181b with c‐Fos) appear to have the strongest effect on CD274 expression highlighting the importance of these miRNAs for future studies and possible therapeutic approaches. This is further underlined by the effect of miR‐155 and other tumour suppressive miRNAs in tumour cells.^14^, ^34^ However, it needs to be taken into account that these miRNAs can also regulate other targets, which might interfere with the therapeutic option. For example, in silico analysis with the TargetScan tool revealed multiple binding sites of miR‐181b on the HLA‐A mRNA, which could negatively interfere with the tumour elimination and might be the case that this miRNA did not influence T cell cytotoxicity.

The constitutive expression of the miRNA candidates identified in this study differs to a certain extent between CD274‐positive and CD274‐negative melanoma lesions, with higher levels in the latter. This finding emphasizes that the miRNA deregulation is most likely required for differential CD274 expression, but this relationship has to be further investigated in patient cohorts with higher variability in CD274 expression This will give further insights into the role of these miRNAs in settings complying to different protocols for CPI eligibility despite thresholds of PD‐L1 are highly variable depending on the antibody used and are not clearly defined.^35^ Moreover, the expression of the miRNA candidates miR‐155 and miR‐186 was consistently found at reduced levels when compared to the other target miRNAs Due to the lack of healthy controls, we could not determine whether the downregulation of miR‐155 and miR186 or the upregulation of the other three candidates could be connected to the initiation and progression of melanoma in general. Additionally, the discrepancy in the miRNA correlation detected could possibly indicate changes in the melanoma cells themselves as well as in the immune infiltrate within the TME that could be connected to the CD274 expression. Since the expression of some miRNAs were positively correlated independent of CD274, common regulatory pathways were hypothesized suggesting that a deregulation of miRNAs resulted in changes of CD274 expression. It is noteworthy that CD274^+^ patients showed a direct correlation of miR‐155, miR‐181b, and miR‐186. All these three miRNAs strongly downregulated CD274 further indicating that these miRNAs should be studied together and not individually, which resulted in the generation of the PDM score. The dynamics between CD274 mRNA and protein expression in combination with the finding that the observed correlations exist further support the importance of these miRNAs as regulators of CD274 expression.

The created PDM scoring system based on the functional efficiency of the identified miRNA candidates on the CD274 surface expression have a very strong prognostic value in our small cohort of melanoma patients. The PDM score appears to be a negative independent prognostic factor along with the T status regardless of other clinicopathological characteristics and despite its correlation with the CD8^+^ T cell infiltration. These data indicate that its prognostic value is not solely to be attributed to the CD274 regulation, but could be linked to additional targets of our miRNA candidates. In vitro as well as in vivo using animal experiments the vast majority of our miRNA targets have been proven to regulate important factors of the immune response.^36^ MiR‐155 has been shown to directly regulate the immune checkpoint molecule CD152 (CTLA‐4),^37^ while miR‐29a and miR‐186 are involved in the JAK/STAT pathway either by direct down regulation of IFN‐γ (miR‐29a) or through SHP‐2(miR‐186),^38^ respectively. Finally, two other CD274 regulating miRNAs affect the TGF‐β pathway with miR‐17 regulating TGFBR2 and miR‐181b indirectly leading to TGF‐β upregulation.^39^ Generally, these miRNAs have been shown to be involved in pro‐ and anti‐inflammatory pathways thereby determining the magnitude of the immune responses. Consequently, most of the miRNAs included in the PDM score have been associated with both oncogenic and tumour suppressive activities in cancer, due to their multifunctional immune regulatory nature.^40^, ^41^, ^42^, ^43^ In tumours, some of these miRNA candidates could have detrimental effects in melanoma progression and metastasis. For example, miR‐155 and miR‐186 have been reported to promote cell proliferation in melanoma by inhibiting the expression of tumour suppressor genes.^44^, ^45^ Similar findings were reported for NSCLC.^46^ However, it is noteworthy miR‐186 has been investigated in various cancer types without a common consensus, due to the large amount of target genes regulated.^43^ On the other hand, miR‐181 and miR‐155 have been suggested as potential negative biomarkers in various cancers.^47^, ^48^ Additionally, the miR‐17‐92 family is known for its oncogenic function and found to be expressed at higher levels in metastatic melanoma,^49^ thus supporting the negative prognostic significance of our scoring system. The connection between CD274, PDM score, and patients’ DFS is prominent on PDM_high_ patients, with CD274^–^ patients demonstrating a better prognosis. This postulates that the negative effects of these upregulated miRNAs are diminished when they actually lead to CD274 downregulation reinforcing the significance of the PDM score and indicating that a differential assessment of the prognostic potential of these miRNAs is necessary, depending on the importance of the CD274‐mediated inhibition in each cancer type.

Finally, the prognostic value of the suggested comPDM signature was increased by combining the PDM score, the CD274 expression as well as the CD8^+^ T cell infiltrate in the melanoma lesions. This was not only used to further stratify PDM_int_ and PDM_high_ patients, but also to underline the possible role of the PDM score in the prediction of CD274 expression. As discussed above, these miRNAs could be involved in additional pathways involved with T cell activity, which could explain why the predictive value of the PDM score is detectable only after cumulative analysis. Furthermore, CD8^+^ T cells have been correlated both with increased overall survival (OS) in metastatic melanoma and an IFN‐γ‐mediated CD274 upregulation, suggesting them as promising candidates for the final model. The comPDM signature stratifies our patients in three distinct groups. In the first group the low PDM score, indicating the reduced expression of our miRNA candidates, allows for increased DFS. As the PDM score increases, patients with intermediate PDM score, but high enough CD8^+^ T cell infiltration accompanied by CD274 expression as well as PDM_high_ patients whose high miRNA levels appear to actively suppress CD274 have slightly worse prognosis. Lastly, the protecting nature of CD8^+^ T cells appears insufficient within PDM_high_CD274^+^ patients. As high expression levels of most of miRNA candidates identified has been shown to correlate with a worse prognosis, the importance of CD8^+^ T cell infiltration is underlined in melanoma lesions, which might benefit from the miRNA‐induced increased proliferation. Thus, poorly infiltrated tumours do not necessarily require high CD274 expression for immune escape.^50^, ^51^ This might be explained next to the general negative impact of the PDM score, by the fact that the high expressed miRNAs could indicate the presence of non‐effector CD8^+^ T cell subtypes, as miRNA expression differs throughout T cell maturation.^52^, ^53^ As a result, these patients together with the PDM_int_ patients without CD8^+^ T cell infiltration were categorized in the comPDM_3_ group, which has the worst prognosis within our small cohort. Consequently, the comPDM signature was generated for the evaluation of patients expressing high levels of the miRNAs identified based on whether this high expression is playing a role in CD274 downregulation or is promoting tumour proliferation, while it has to be also taken into account that the miRNA‐mediated CD274 suppression can have a beneficial based on CD8^+^ T cell infiltration. These data suggest an interplay between miRNAs, CD274, and the immune infiltrate, which might allow us to better optimize potential miRNA therapies, to be used as a single therapy or in combination with CPI. The involvement of these miRNAs in the regulation of various pro‐ and anti‐oncogenic pathways in both immune and tumour cells indicate that the generation of combinatorial functional signatures is essential not only for improved prognosis, but also for the assessment of therapeutic options with the minimum number of drawbacks for the patient. For example, a chimeric antigen receptor (CAR) T cell could be utilized in this context either by potentially using a 4^th^ generation TRUCK CAR‐T cell model, transferring the expressed miRNAs directly to the tumour site^54^ or by loading PD‐L1 suppressing miRNAs on CAR‐T produced exosomes for increased homing and lower patient toxicity.^55^ Using this approach, an effective delivery system is combined with the cytotoxic activity of CAR T cells. As a result, after consideration of biomarkers, such as the proposed comPDM score of this study, this strategy will allow a simultaneous targeting of different inhibitory receptors due to the affinity of the miRNAs for multiple targets, such as, for example, miR‐155, affecting both PD‐L1 and CTLA‐4.^13^, ^56^ The importance of miRNAs directed against immune checkpoints is further underlined by a study demonstrating a defective anti‐tumour immunity in T cell‐specific miR‐155‐deficient mice.^29^ However, due to the small number of patients examined, the prognostic significance of the PDM score and the comPDM signature has to be validated on larger cohorts of patients.

## 5 CONCLUSION

The present study revealed that the CDS, and not only 3′ UTR targeting microRNAs, affect PD‐L1 expression and function and could serve as prognostic marker and therapeutic targets for melanoma.

## CONFLICT OF INTEREST

The authors declare that there is no conflict of interest that could be perceived as prejudicing the impartiality of the research reported.

## Supporting information

Supporting information

Supporting information

Supporting information

Supporting information

Supporting information

Supporting information

Supporting information

Supporting information

Supporting Information Table 1 – List of the primers used for cDNA synthesis and miRNA expression analysis through qPCR
Supporting Information Table 2 – Selected list of the miRNAs found to target CD274 CDS and 3′‐UTR through RNAseq analysis after miTRAP. The absolute counts of the miRNA enrichment in the targeted (CDS or 3′‐UTR) and the control MS2 loop sequence are presented along with the fold enrichment of the miRNAs (ratio of enrichment in target sequence/enrichment in MS2 loop sequence) and the mature sequences of the miRNAs in question. Only the miRNAs that fit our selection criteria are shown.
Supporting Information Table 3 – Cumulative list of the sequencing reads (target sequence and MS2 sequence) of all the miRNAs detected through RNAseq after miTRAP along with their fold enrichment.
