# Supplementary material for: Identification and characterization of novel CD274 (PD‐L1) regulating microRNAs and their functional relevance in melanoma
Source: Clin Transl Med. 2022 Jul 8;12(7):e934. doi: 10.1002/ctm2.934 (PMC9270002; doi:10.1002/ctm2.934)
Supplement: Supplementary file 1 — Supporting information [file CTM2-12-e934-s004.pdf]

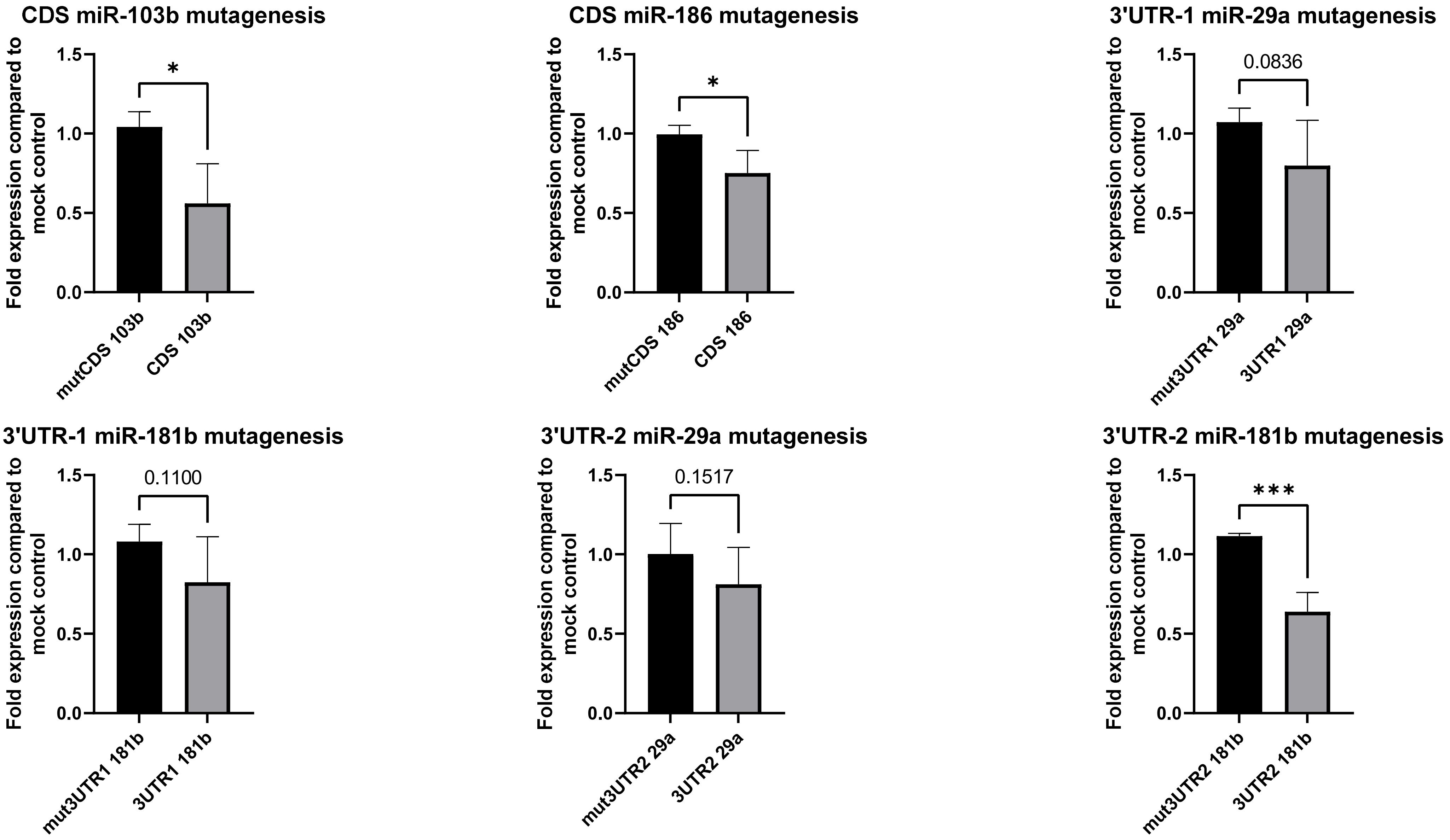

CDS miR-103b mutagenesis
Fold expression compared to mock control
1.5
1.0
0.5
0.0
*
mutCDS 103b
CDS 103b
CDS miR-186 mutagenesis
Fold expression compared to mock control
1.5
1.0
0.5
0.0
*
mutCDS 186
CDS 186
3'UTR-1 miR-29a mutagenesis
Fold expression compared to mock control
1.5
1.0
0.5
0.0
0.0836
mut3UTR1 29a
3UTR1 29a
3'UTR-1 miR-181b mutagenesis
Fold expression compared to mock control
1.5
1.0
0.5
0.0
0.1100
mut3UTR1 181b
3UTR1 181b
3'UTR-2 miR-29a mutagenesis
Fold expression compared to mock control
1.5
1.0
0.5
0.0
0.1517
mut3UTR2 29a
3UTR2 29a
3'UTR-2 miR-181b mutagenesis
Fold expression compared to mock control
1.5
1.0
0.5
0.0
***
mut3UTR2 181b
3UTR2 181b
