# Supplementary material for: Identification and characterization of novel CD274 (PD‐L1) regulating microRNAs and their functional relevance in melanoma
Source: Clin Transl Med. 2022 Jul 8;12(7):e934. doi: 10.1002/ctm2.934 (PMC9270002; doi:10.1002/ctm2.934)
Supplement: Supplementary file 2 — Supporting information [file CTM2-12-e934-s006.pdf]

A

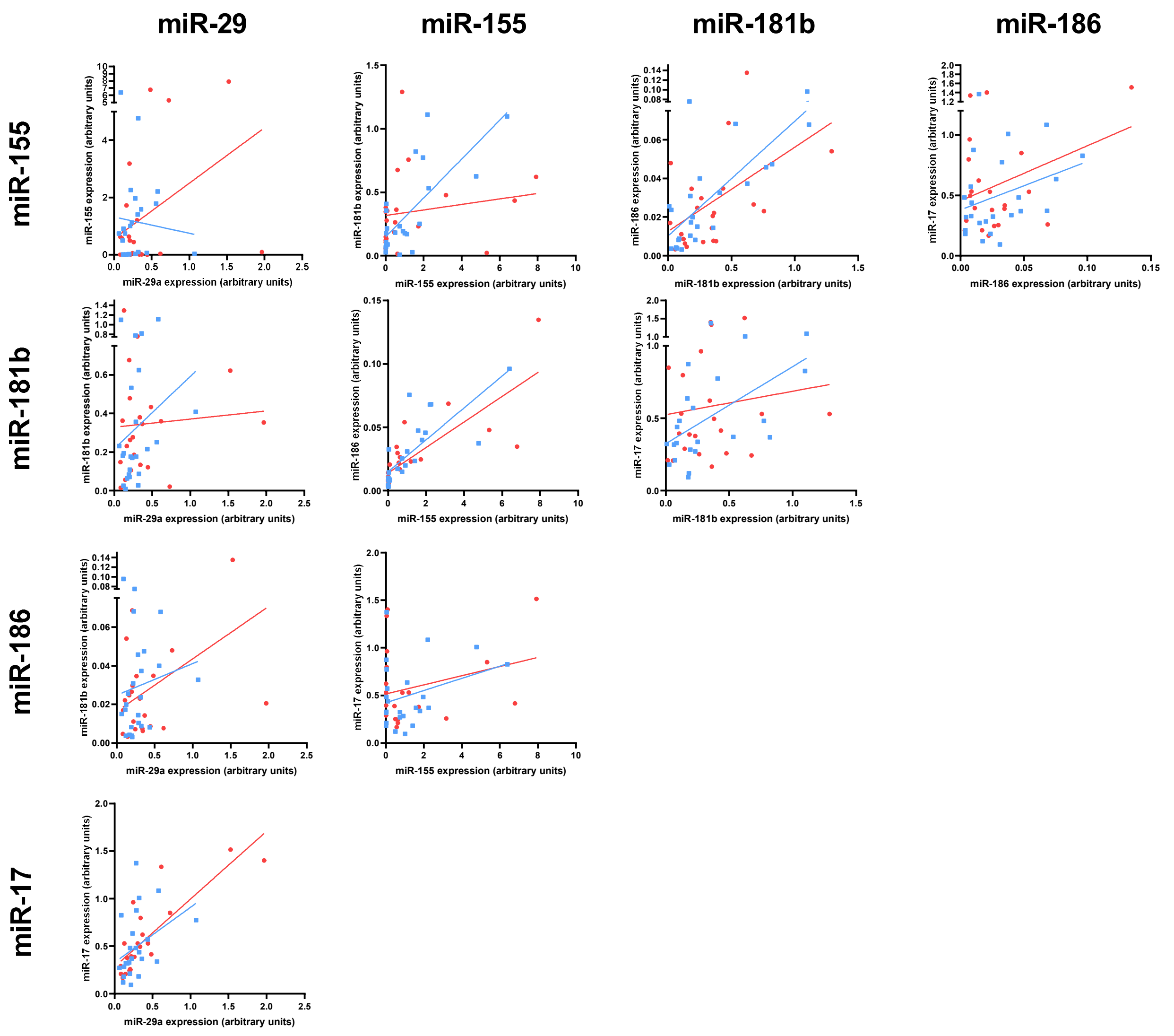

B

CD274-	miR155	miR181b	miR186	miR17
miR-29a	**r=0.389 p=0.073**	r=0.067 p=0.768	**r=0.431 p=0.045**	**r=0.826 p<0.000**
miR-155		r=0.172 p=0.444	**r=0.792 p<0.000**	r=0.277 p=0.212
miR-181b			**r=0.434 p=0.043**	r=0.118 p=0. 602
miR-186				r=0.333 p=0.130

CD274+	miR155	miR181b	miR186	miR17
miR-29a	r=0.234 p=0.271	**r=0.362 p=0.082**	r=0.253 p=0.233	**r=0.530 p=0.008**
miR-155		**r=0.672 p<0.000**	**r=0.900 p<0.000**	r=0.259 p=0.221
miR-181b			**r=0.672 p<0.000**	**r=0.577 p=0.003**
miR-186				r=0.337 p=0.108
