# Supplementary material for: Identification and characterization of novel CD274 (PD‐L1) regulating microRNAs and their functional relevance in melanoma
Source: Clin Transl Med. 2022 Jul 8;12(7):e934. doi: 10.1002/ctm2.934 (PMC9270002; doi:10.1002/ctm2.934)

A

miRNAs	miRNA effect on surface CD274 expression	miRNA maximum point worth
miR-29a	+	1
miR-155	+++	3
miR-181b	++	2
miR-186	+++	3
miR-17	+++	3

Every + equals to a decrease of 10%

B

miRNA expression distribution	Expression multiplier	Individual miRNA score
Q1	0	0
Low Q2 (25%-median)	1/3	1/3*miRNAworth
High Q2 (median-75%)	2/3	2/3*miRNAworth
Q3	1	miRNAworth

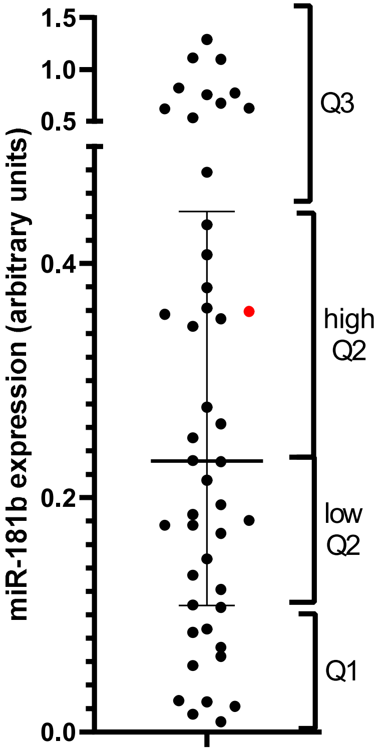

C

Patient PDM score= sum of individual miRscores

$PDM\ score_{min}$=0

$PDM\ score_{max}$=12

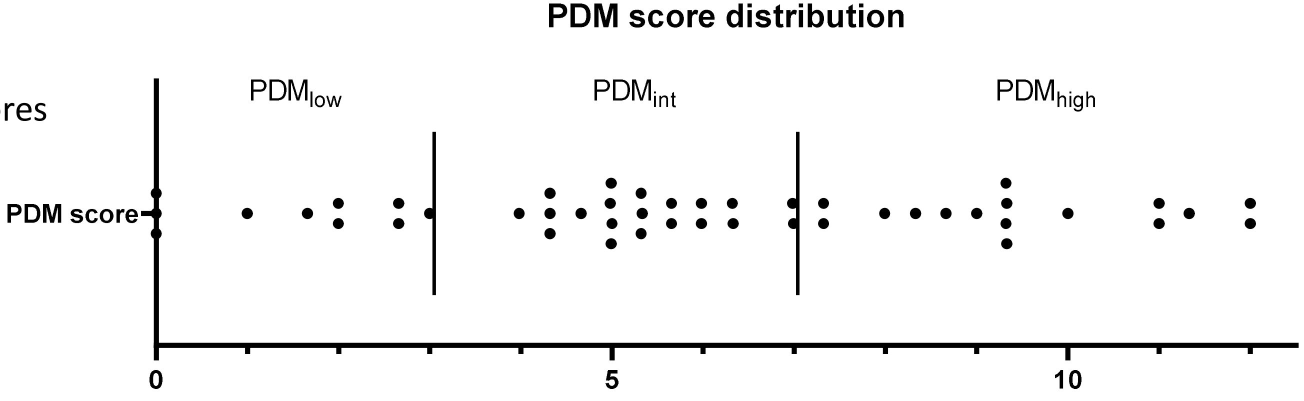

Supplement: Supplementary file 3 — Supporting information [file CTM2-12-e934-s001.pdf]
