# Supplementary figures and images for: Identification and characterization of novel CD274 (PD‐L1) regulating microRNAs and their functional relevance in melanoma
Source: Clin Transl Med. 2022 Jul 8;12(7):e934. doi: 10.1002/ctm2.934 (PMC9270002; doi:10.1002/ctm2.934)

A

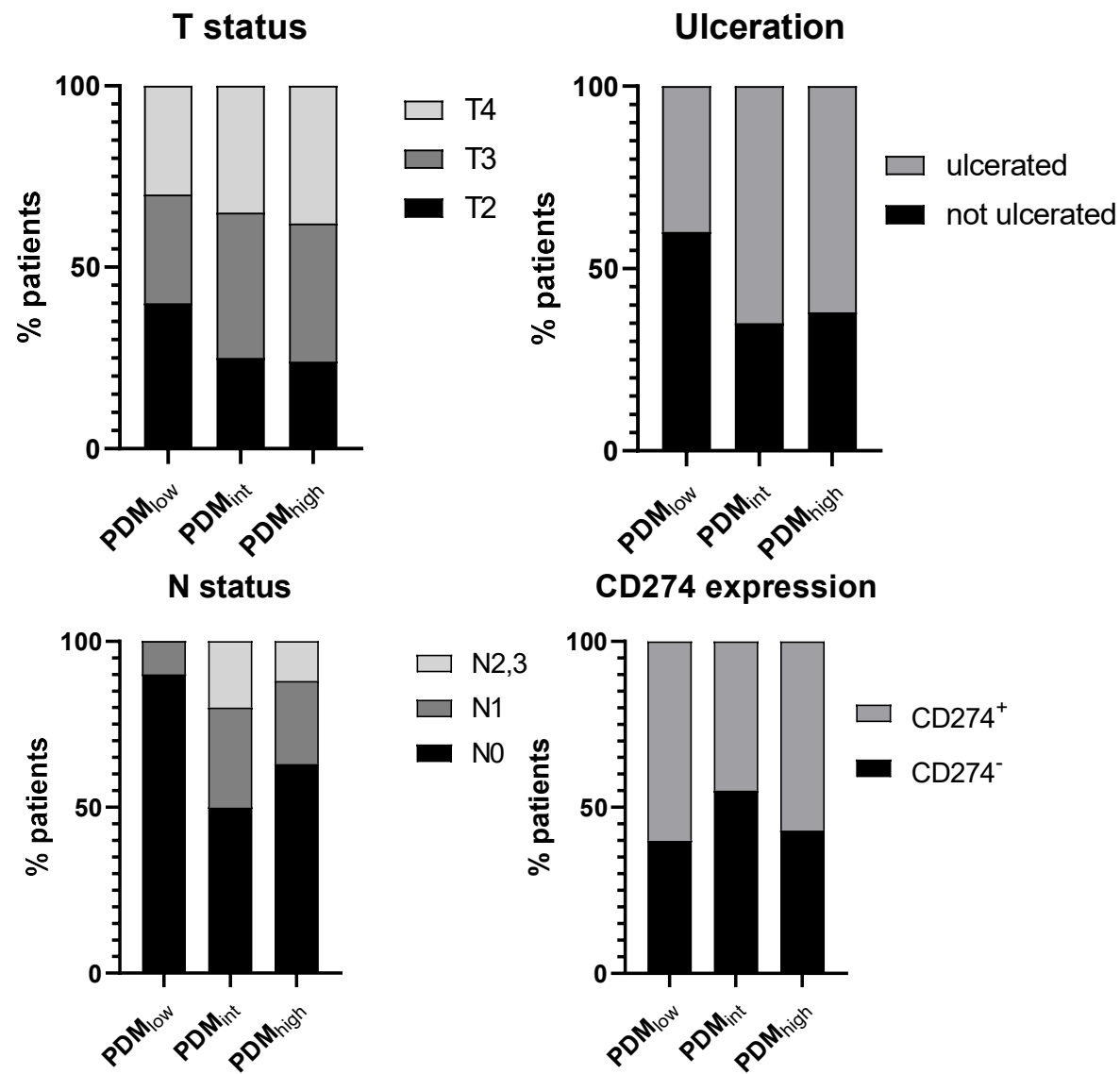

B

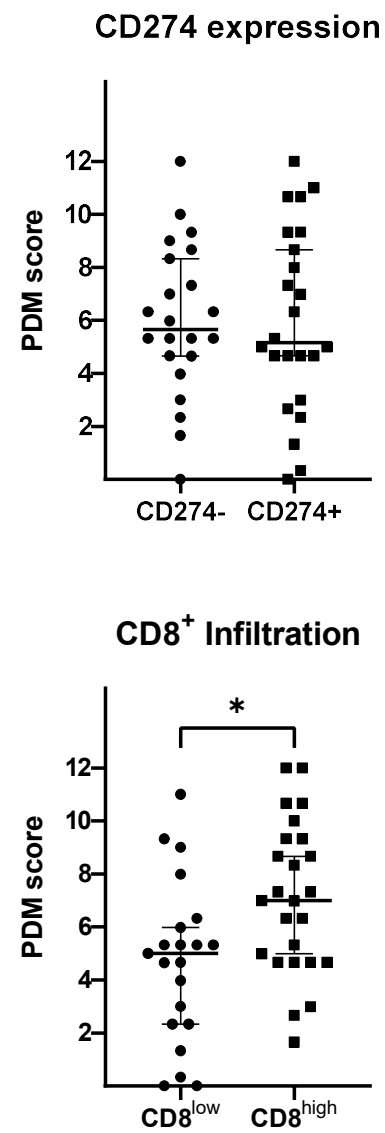

Supplement: Supplementary file 4 — Supporting information [file CTM2-12-e934-s005.pdf]
