# Supplementary material for: Identification and characterization of novel CD274 (PD‐L1) regulating microRNAs and their functional relevance in melanoma
Source: Clin Transl Med. 2022 Jul 8;12(7):e934. doi: 10.1002/ctm2.934 (PMC9270002; doi:10.1002/ctm2.934)
Supplement: Supplementary file 5 — Supporting information [file CTM2-12-e934-s009.pdf]

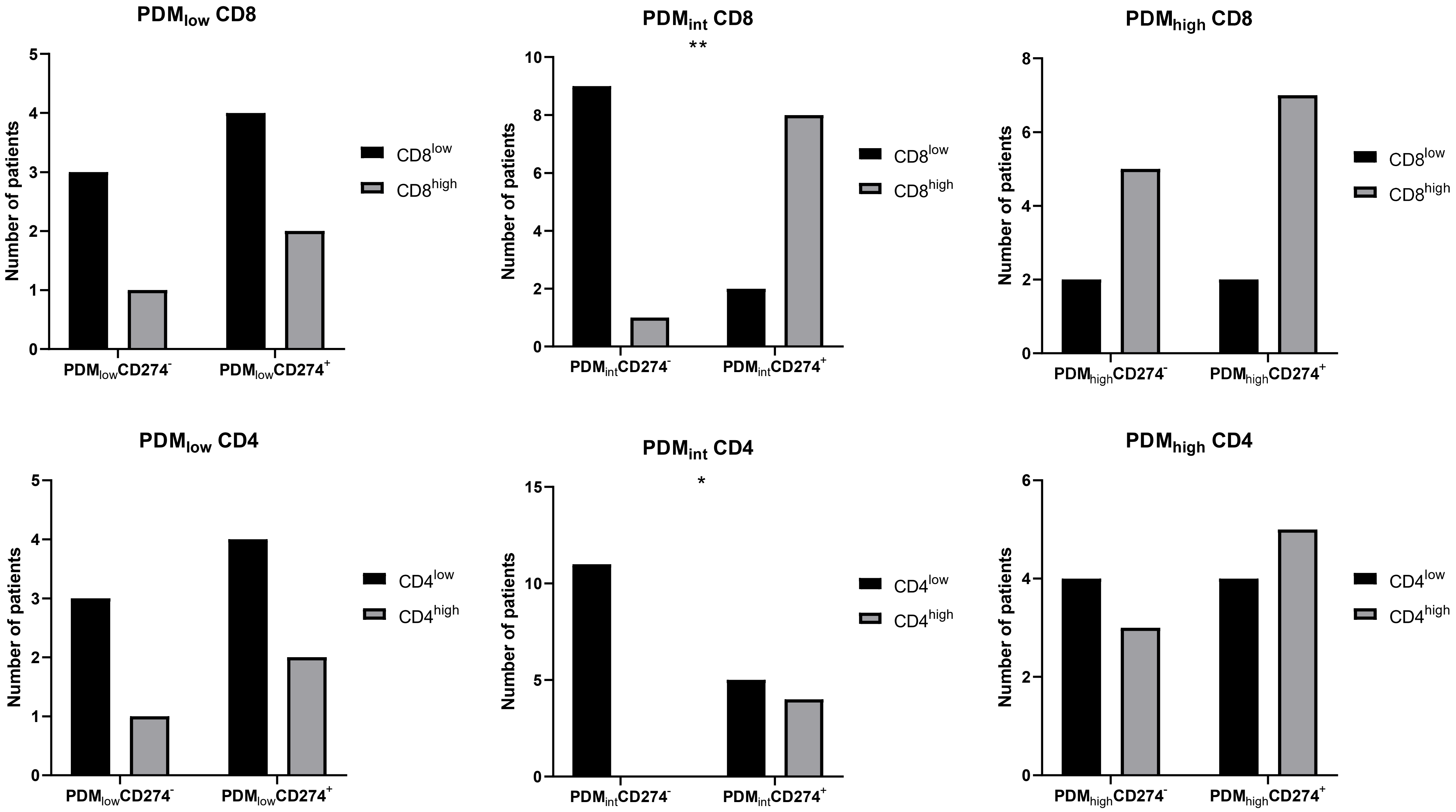

PDMlow CD8
Number of patients
0
1
2
3
4
5
CD8low
CD8high
PDMlowCD274-
PDMlowCD274+
PDMint CD8
**
0
2
4
6
8
10
PDMintCD274-
PDMintCD274+
PDMhigh CD8
0
2
4
6
8
PDMhighCD274-
PDMhighCD274+
PDMlow CD4
CD4low
CD4high
PDMint CD4
*
0
5
10
15
PDMhigh CD4
0
2
4
6
