# Supplementary material for: Identification and characterization of novel CD274 (PD‐L1) regulating microRNAs and their functional relevance in melanoma
Source: Clin Transl Med. 2022 Jul 8;12(7):e934. doi: 10.1002/ctm2.934 (PMC9270002; doi:10.1002/ctm2.934)
Supplement: Supplementary file 6 — Supporting information [file CTM2-12-e934-s003.pdf]

primer name	sequence	primer name	sequence
miR-0009-5p qPCR FW	GCCCGCTCTTTGGTTATCTAGC	miR-0148b-3p qPCR FW	GCCCTCAGTGCATCACAGAAC
miR-0009-5p SL RT	GTCGTATCCAGTGCAGGGTCCGAGGTATTCGCACTGGATACGAC TCATAC	miR-0152-3p qPCR FW	GCCCTCAGTGCATGACAGA
miR-0015a-5p qPCR FW	GCCCTAGCAGCACATAATGG	miR-0152-3p RT SL	GTCGTATCCAGTGCAGGGTCCGAGGTATTCGCACTGGATACGAC CCAAGT
miR-0015a-5p RT SL	GTCGTATCCAGTGCAGGGTCCGAGGTATTCGCACTGGATACGAC CACAAA	miR-0155-5p qPCR FW	CACGCATTAATGCTAATCGTGAT
miR-0016-5p qPCR FW	GCCCCTAGCAGCACGTAAATA	miR-0155-5p SL RT	TCGTATCCAGTGCAGGGTCCGAGGTATTCGCACTGGATACGAC ACCCCT
miR-0016-5p SL RT	GTCGTATCCAGTGCAGGGTCCGAGGTATTCGCACTGGATACGAC CGCCAA	miR-0181a-5p qPCR FW	CACGCAAACATTCAACGCTGTC
miR-0017-5p qPCR FW	GCCCCAAAGTGCTTACAGTG	miR-0181a-5p SL RT	TCGTATCCAGTGCAGGGTCCGAGGTATTCGCACTGGATACGAC ACTCAC
miR-0017-5p RT SL	GTCGTATCCAGTGCAGGGTCCGAGGTATTCGCACTGGATACGAC CTACCT	miR-0181b-5p qPCR FW	GCCCAACATTCATTGCTGTC
miR-0025-3p qPCR FW	GCCCCATTGCACTTGTCTCG	miR-0181b-5p RT SL	GTCGTATCCAGTGCAGGGTCCGAGGTATTCGCACTGGATACGAC ACCCAC
miR-0025-3p RT SL	GTCGTATCCAGTGCAGGGTCCGAGGTATTCGCACTGGATACGAC TCAGAC	miR-0186-5p qPCR FW	GCCCCAAAGAATTCTCCTTT
miR-0026a RT SL	GTCGTATCCAGTGCAGGGTCCGAGGTATTCGCACTGGATACGAC AGCCTA	miR-0186-5p RT SL	GTCGTATCCAGTGCAGGGTCCGAGGTATTCGCACTGGATACGAC AGCCCA
miR-0026a-5p qPCR FW	GCCCTTCAAGTAATCCAGGA	miR-0200c-3p qPCR FW	GCCCTAATACTGCCGGGTAA
miR-0026b-5p SL RT	GTCGTATCCAGTGCAGGGTCCGAGGTATTCGCACTGGATACGAC ACCTAT	miR-0200c-3p RT SL	GTCGTATCCAGTGCAGGGTCCGAGGTATTCGCACTGGATACGAC TCCATC
miR-0027a-3p qPCR FW	CACGCATTCACAGTGGCTAAG	miR-0320a SL RT	GTCGTATCCAGTGCAGGGTCCGAGGTATTCGCACTGGATACGAC TCGCCC
miR-0027a-3p SL RT	GTCGTATCCAGTGCAGGGTCCGAGGTATTCGCACTGGATACGAC GCGGAA	miR-0320a/b qPCR FW	GCCAAAAGCTGGGTTGAGAG
miR-0027b-3p qPCR FW	GCCCTTCACAGTGGCTAAGT	miR-0324-5p qPCR FW	GAAACGCATCCCCTAGGGCAT
miR-0027b-3p RT SL	GTCGTATCCAGTGCAGGGTCCGAGGTATTCGCACTGGATACGAC GCAGAA	miR-0324-5p RT SL	GTCGTATCCAGTGCAGGGTCCGAGGTATTCGCACTGGATACGAC ACACCA
miR-0029a-3p qPCR FW	GCCCTAGCACCATCTGAAAT	miR-0340-5p qPCR FW	GCCCGGTTATAAAGCAATGAGAC
miR-0029a-3p RT SL	GTCGTATCCAGTGCAGGGTCCGAGGTATTCGCACTGGATACGAC TAACCG	miR-0340-5p RT SL	GTCGTATCCAGTGCAGGGTCCGAGGTATTCGCACTGGATACGAC AATCAG
miR-0029b qPCR FW	GCCCTAGCACCATTTGAAATC	miR-0424-5p qPCR FW	CACGCACAGCAGCAATTCATG
miR-0029b RT SL	GTCGTATCCAGTGCAGGGTCCGAGGTATTCGCACTGGATACGAC AACACT	miR-0424-5p SL RT	TCGTATCCAGTGCAGGGTCCGAGGTATTCGCACTGGATACGAC TTCAAA
miR-0034a qPCR FW	GCCCCAATCAGCAAGTATACTGC	miR-0516b-5p qPCR FW	GCGCAATCTGGAGGTAAGAAGC
miR-0034a RT SL	GTCGTATCCAGTGCAGGGTCCGAGGTATTCGCACTGGATACGAC AGGGCA	miR-0516b-5p SL RT	GTCGTATCCAGTGCAGGGTCCGAGGTATTCGCACTGGATACGAC AAAGTG
miR-0034a-5p qPCR FW	GCCCTGGCAGTGTCTTAG	SL qPCR RV	CCAGTGCAGGGTCCGAGGTA
miR-0034a-5p SL RT	GTCGTATCCAGTGCAGGGTCCGAGGTATTCGCACTGGATACGAC ACAACC	U6 snRNA qPCR FW	CGGCAGCACATATACTAAAATTGGA
miR-0103a-3p qPCR FW	GCCCAGCAGCATTGTACAGG	U6 snRNA qPCR RV	AATATGGAACGCTTCACGAATTTGC
miR-0103a-3p RT SL	GTCGTATCCAGTGCAGGGTCCGAGGTATTCGCACTGGATACGAC TCATAG	RNU44 FW	CCTGGATGATGATAAGCAAATGC
miR-0103b-5p qPCR FW	GCCCTCATAGCCCTGTACAA	RNU44 RV	CAGTTAGAGCTAATTAAGACCTTC
miR-0103b-5p RT SL	GTCGTATCCAGTGCAGGGTCCGAGGTATTCGCACTGGATACGAC AGCAGC	RNU46 FW	GGGTGATGAAAAAGAATCCTTAGG
miR-0146a-5p qPCR FW	CACGCATGAGAACTGAATTCC	RNU46 RV	GTGTAACTATGACAAGTCCTTGC
miR-0146a-5p SL RT	GTCGTATCCAGTGCAGGGTCCGAGGTATTCGCACTGGATACGAC AACCCA	RNU47 FW	GATGTAATGATTCTGCCAAATG
miR-0148a-3p qPCR FW	GCCCTCAGTGCACTACAGAAC	RNU47 RV	CCTCAGAATCAAAATGGAACG
miR-0148a/b-3p RT SL	GTCGTATCCAGTGCAGGGTCCGAGGTATTCGCACTGGATACGAC ACAAAG		
