# Supplementary material for: Identification and characterization of novel CD274 (PD‐L1) regulating microRNAs and their functional relevance in melanoma
Source: Clin Transl Med. 2022 Jul 8;12(7):e934. doi: 10.1002/ctm2.934 (PMC9270002; doi:10.1002/ctm2.934)
Supplement: Supplementary file 7 — Supporting information [file CTM2-12-e934-s008.pdf]

CDS

miRNA	CDS	MS2	fold enrichment	sequence
hsa-miR-584-5p	9834.6	114.9	85.6	UUAUGGUUUGCCUGGGACUGAG
hsa-miR-9-5p	15423.9	206.9	74.6	UCUUUGGUUAUCUAGCUGUAUGA
hsa-miR-550a-3-5p	1429.3	23	62.2	AGUGCCUGAGGGAGUAAGAG
hsa-miR-550a-5p	1429.3	23	62.2	AGUGCCUGAGGGAGUAAGAGCCC
hsa-miR-454-3p	1898.7	46	41.3	UAGUGCAAUAUUGCUUAUAGGGU
hsa-miR-27b-3p	69311.7	1792.8	38.7	UUCACAGUGGCUAAGUUCUGC
hsa-let-7g-5p	14677.3	436.7	33.6	UGAGGUAGUAGUUUGUACAGUU
hsa-miR-181a-5p	6720	206.9	32.5	AACAUUCAACGCUGUCGGUGAGU
hsa-miR-122-5p	1685.3	69	24.4	UGGAGUGUGACAAUGGUGUUUG
hsa-miR-3591-3p	1685.3	69	24.4	AAACACCAUUGUCACACUCCAC
hsa-miR-20a-5p	7829.3	321.8	24.3	UAAAGUGCUUAUAGUGCAGGUAG
hsa-miR-27a-3p	51626.4	2413.4	21.4	UUCACAGUGGCUAAGUUCCGC
hsa-miR-103a-3p	23039.9	1172.2	19.7	AGCAGCAUUGUACAGGGCUAUGA
hsa-miR-185-3p	1173.3	69	17	AGGGGCUGGCUUUCCUCUGGUC
hsa-miR-25-5p	1514.7	91.9	16.5	AGGCGGAGACUUGGGCAAUUG
hsa-miR-222-3p	18858.6	1402.1	13.5	AGCUACAUCUGGCUACUGGGU
hsa-miR-629-5p	4608	344.8	13.4	UGGGUUUACGUUGGGAGAACU
hsa-miR-4521	8960	735.5	12.2	GCUAAGGAAGUCCUGUGCUCAG
hsa-let-7e-5p	5781.3	482.7	12	UGAGGUAGGAGGUUGUAUAGUU
hsa-miR-211-5p	2773.3	252.8	11	UUCCCUUUGUCAUCCUUCGCCU
hsa-miR-221-3p	7829.3	758.5	10.3	AGCUACAUUGUCUGCUGGGUUUC
hsa-let-7f-5p	10773.3	1264.2	8.5	UGAGGUAGUAGAUUGUAUAGUU
hsa-miR-128-3p	3626.7	505.7	7.2	UCACAGUGAACCGGUCUCUUU
hsa-miR-93-5p	2538.7	367.8	6.9	CAAAGUGCUGUUCGUGCAGGUAG
hsa-let-7i-5p	10602.6	1563	6.8	UGAGGUAGUAGUUUGUGCUGUU
hsa-let-7c-5p	3840	597.6	6.4	UGAGGUAGUAGGUUGUAUGGUU
hsa-miR-23a-3p	1834.7	390.7	4.7	AUCACAUUGCCAGGGAUUUCC
hsa-miR-378a-3p	4373.3	1011.3	4.3	ACUGGACUUGGAGUCAGAAGGC
hsa-miR-105-5p	1152	298.8	3.9	UCAAAUGCUCAGACUCCUGUGGU
hsa-let-7b-5p	7040	2183.6	3.2	UGAGGUAGUAGGUUGUGUGGUU
hsa-miR-193a-5p	4458.6	1448.1	3.1	UGGGUCUUUGCGGGCGAGAUGA
hsa-miR-451a	1770.7	620.6	2.9	AAACCGUUACCAUUACUGAGUU
hsa-miR-103a-2-5p	1130.7	0	-	AGCUUCUUUACAGUGCUGCCUUG
hsa-miR-550b-3p	1237.3	0	-	UCUUACUCCCUCAGGCACUG

3’-UTR-1

miRNA	3'UTR-1	MS2	fold enrichment	sequence
hsa-miR-140-5p	26822.2	207.7	129.1	CAGUGGUUUUACCCUAUGGUAG
hsa-miR-425-5p	6937.1	115.4	60.1	AAUGACACGAUCACUCCCGUUGA
hsa-miR-30b-5p	1312.8	23.1	56.9	UGUAAACAUCCUACACUCAGCU
hsa-miR-574-3p	2125.8	92.3	23	CACGCUCAUGCACACACCCACA
hsa-miR-29b-3p	1759.3	92.3	19.1	UAGCACCAUUUGAAAUCAGUGUU
hsa-miR-9-5p	3485.2	207.7	16.8	UCUUUGGUUAUCUAGCUGUAUGA
hsa-miR-320b	9669.3	692.4	14	AAAAGCUGGGUUGAGAGGGCAA
hsa-miR-25-3p	6217.4	623.1	10	CAUUGCACUUGUCUCGGUCUGA
hsa-miR-103a-3p	11675.2	1177	9.9	AGCAGCAUUGUACAGGGCUAUGA
hsa-miR-320c	1572.7	184.6	8.5	AAAAGCUGGGUUGAGAGGGU
hsa-miR-629-5p	2419	346.2	7	UGGGUUUACGUUGGGAGAACU
hsa-miR-24-3p	17179.6	3208	5.4	UGGCUCAGUUCAGCAGGAACAG
hsa-miR-3074-5p	16853	3184.9	5.3	GUUCCUGCUGAACUGAGCCAG
hsa-miR-532-3p	1186.2	230.8	5.1	CCUCCCACACCCAAGGCUUGCA
hsa-miR-181a-5p	1039.6	207.7	5	AACAUUCAACGCUGUCGGUGAGU
hsa-miR-27b-3p	7223.7	1800.2	4	UUCACAGUGGCUAAGUUCUGC
hsa-miR-30c-5p	5890.9	1546.3	3.8	UGUAAACAUCCUACACUCUCAGC
hsa-miR-151a-3p	5977.5	1707.9	3.5	CUAGACUGAAGCUCCUUGAGG
hsa-miR-4521	2199.1	738.5	3	GCUAAGGAAGUCCUGUGCUCAG
hsa-miR-106b-3p	1572.7	530.8	3	CCGCACUGUGGGUACUUGCUGC
hsa-miR-23a-3p	1126.2	392.3	2.9	AUCACAUUGCCAGGGAUUUCC

3’-UTR-2

miRNA	3'UTR-2	MS2	fold enrichment	sequence
hsa-miR-20a-5p	40453.9	94.7	427.4	UAAAGUGCUUAUAGUGCAGGUAG
hsa-miR-146b-5p	7683.5	74.4	103.3	UGAGAACUGAAUUCCAUAGGCUG
hsa-miR-3074-5p	11949.7	933.1	12.8	GUUCCUGCUGAACUGAGCCAG
hsa-miR-24-3p	12011	939.8	12.8	UGGCUCAGUUCAGCAGGAACAG
hsa-miR-151a-3p	4772.8	500.4	9.5	CUAGACUGAAGCUCCUUGAGG
hsa-miR-30e-5p	1281.1	209.6	6.1	UGUAAACAUCCUUGACUGGAAG
hsa-miR-103a-3p	1190.8	344.8	3.5	AGCAGCAUUGUACAGGGCUAUGA
hsa-miR-222-3p	1394.1	412.5	3.4	AGCUACAUCUGGCUACUGGGU
hsa-miR-340-5p	364.7	108.2	3.4	UUAUAAAGCAAUGAGACUGAUU
