# Supplementary material for: Identification and characterization of novel CD274 (PD‐L1) regulating microRNAs and their functional relevance in melanoma
Source: Clin Transl Med. 2022 Jul 8;12(7):e934. doi: 10.1002/ctm2.934 (PMC9270002; doi:10.1002/ctm2.934)
Supplement: Supplementary file 8 — Supporting information [file CTM2-12-e934-s002.pdf]

CDS

miRNA	CDS	MS2 control	enrichment CDS/MS2
hsa-miR-148b-3p	77098,34089	183,8795703	419,2871496
hsa-miR-584-5p	9834,62511	114,9247314	85,57448853
hsa-miR-9-5p	15423,93483	206,8645165	74,56056304
hsa-miR-550a-3-5p	1429,327294	22,98494628	62,18536585
hsa-miR-550a-5p	1429,327294	22,98494628	62,18536585
hsa-miR-454-3p	1898,658644	45,96989257	41,3022206
hsa-miR-27b-3p	69311,70712	1792,82581	38,66059197
hsa-let-7g-5p	14677,27131	436,7139794	33,60843025
hsa-miR-181a-5p	6719,971605	206,8645165	32,48489261
hsa-miR-34a-5p	618,6640525	22,98494628	26,91605388
hsa-miR-148a-3p	257598,9115	10343,22583	24,90508433
hsa-miR-122-5p	1685,326212	68,95483885	24,44101444
hsa-miR-3591-3p	1685,326212	68,95483885	24,44101444
hsa-miR-20a-5p	7829,300251	321,789248	24,33052161
hsa-miR-107	533,3310797	22,98494628	23,20349472
hsa-miR-27a-3p	51626,44852	2413,41936	21,39141228
hsa-miR-181b-5p	7658,634305	367,7591405	20,82513651
hsa-miR-152-3p	3306,652694	160,894624	20,55166675
hsa-miR-103a-3p	23039,90264	1172,23226	19,65472494
hsa-miR-103b	22207,90616	1149,247314	19,3238704
hsa-miR-185-3p	1173,328375	68,95483885	17,01589613
hsa-miR-25-5p	1514,660266	91,93978513	16,47448125
hsa-miR-17-5p	4330,648368	275,8193554	15,70103143
novel_39	3903,983504	275,8193554	14,15413178
hsa-miR-222-3p	18858,58698	1402,081723	13,45041924
hsa-miR-629-5p	4607,980529	344,7741942	13,36521296
hsa-miR-4521	8959,96214	735,518281	12,18183473
hsa-let-7e-5p	5781,308904	482,6838719	11,97742299
hsa-miR-19b-3p	533,3310797	45,96989257	11,60174736
hsa-miR-211-5p	2773,321615	252,8344091	10,96892478
hsa-miR-221-3p	7829,300251	758,5032273	10,32203947
hsa-miR-486-5p	874,6629708	91,93978513	9,513432836
hsa-miR-486-3p	853,3297276	91,93978513	9,281397889
hsa-let-7f-5p	10773,28781	1264,172046	8,522010789
hsa-let-7a-5p	90538,2841	11630,38282	7,784634909
hsa-miR-23b-3p	511,9978366	68,95483885	7,425118311
hsa-miR-128-3p	3626,651342	505,6688182	7,171989278
hsa-miR-93-5p	2538,65594	367,7591405	6,90303968
hsa-let-7i-5p	10602,62187	1562,976347	6,783609927
hsa-let-7c-5p	3839,983774	597,6086033	6,425583154
hsa-miR-199a-3p	426,6648638	68,95483885	6,187598592
hsa-miR-23a-3p	1834,658914	390,7440868	4,695295402
hsa-miR-378a-3p	4373,314854	1011,337636	4,324287653
hsa-miR-191-5p	14357,27267	3516,696781	4,082601816
hsa-miR-378c	447,998107	114,9247314	3,898187113
hsa-miR-105-5p	1151,995132	298,8043017	3,855349892
hsa-miR-455-5p	682,6637821	206,8645165	3,300052583
hsa-let-7b-5p	7039,970253	2183,569897	3,22406453
hsa-miR-193a-5p	4458,647827	1448,051616	3,079066919
hsa-miR-451a	1770,659185	620,5935496	2,853170462
hsa-miR-27a-5p	2559,989183	1057,307529	2,421234232
hsa-miR-103a-2-5p	1130,661889	0	n.d.
hsa-miR-181d-5p	426,6648638	0	n.d.
hsa-miR-550b-3p	1237,328105	0	n.d.

3’UTR 2

miRNA	3'UTR-2	MS2	enrichment 3'-1/MS2
hsa-miR-34a-5p	18084,22	6,761493	2674,590015
hsa-miR-106b-5p	20181,78	13,52299	1492,405956
hsa-miR-17-5p	37559,29	81,13791	462,906751
hsa-miR-20a-5p	40453,92	94,6609	427,3562307
hsa-miR-146a-5p	3142182	18533,25	169,5429311
hsa-miR-93-5p	15554,24	108,1839	143,7759176
hsa-miR-146b-5p	7683,535	74,37642	103,306061
hsa-miR-107	203,3023	6,761493	30,06766077
hsa-miR-20b-5p	458,2369	20,28448	22,59051762
hsa-miR-18a-5p	287,2048	13,52299	21,23826832
hsa-miR-26b-5p	661,5391	33,80746	19,56784273
hsa-miR-449c-5p	258,1616	13,52299	19,09057827
hsa-miR-454-3p	235,5725	13,52299	17,42015267
hsa-miR-221-5p	858,3874	60,85343	14,10581617
hsa-miR-148b-3p	758,3497	54,09194	14,01964342
hsa-miR-3074-5p	11949,66	933,086	12,80659626
hsa-miR-24-3p	12010,97	939,8475	12,77970006
hsa-miR-629-5p	993,9222	101,4224	9,799830178
hsa-miR-151a-3p	4772,763	500,3505	9,538839615
hsa-miR-181a-5p	561,5015	60,85343	9,22711283
hsa-miR-140-5p	555,0475	60,85343	9,121054062
hsa-miR-181b-5p	893,8846	108,1839	8,262640907
hsa-miR-25-5p	212,9833	27,04597	7,874863536
hsa-miR-23a-3p	861,6144	114,9454	7,495859408
hsa-miR-652-3p	145,2159	20,28448	7,158966851
hsa-miR-769-5p	141,9889	20,28448	6,999878698
hsa-miR-26a-5p	22340,66	3292,847	6,784603355
hsa-miR-514a-3p	271,0697	40,56896	6,681702394
hsa-miR-7641	513,0962	81,13791	6,323754051
hsa-miR-30e-5p	1281,127	209,6063	6,112064171
hsa-miR-509-3p	112,9457	20,28448	5,568085328
hsa-miR-516b-5p	96,81061	20,28448	4,772644567
hsa-miR-105-5p	396,9235	87,89941	4,515656014
hsa-miR-28-5p	167,805	40,56896	4,136291958
hsa-miR-486-5p	109,7187	27,04597	4,056747882
hsa-miR-486-3p	106,4917	27,04597	3,937431768
hsa-miR-135b-5p	203,3023	54,09194	3,758457597
hsa-miR-584-5p	122,6268	33,80746	3,627209871
hsa-miR-103a-3p	1190,77	344,8361	3,453148716
hsa-miR-222-3p	1394,073	412,4511	3,379971234
hsa-miR-340-5p	364,6533	108,1839	3,370680226
hsa-miR-103b	1135,911	338,0746	3,359941775
hsa-miR-30a-5p	897,1116	283,9827	3,159036166
hsa-miR-148a-3p	8125,637	3042,672	2,670559782
hsa-miR-27b-5p	70,99444	27,04597	2,624954512
hsa-miR-451a	416,2856	182,5603	2,280263515
hsa-miR-1268a	238,7995	108,1839	2,207348112
hsa-miR-152-3p	96,81061	47,33045	2,0454191
hsa-miR-532-5p	667,9932	331,3131	2,016198827
hsa-miR-23a-5p	67,76742	33,80746	2,004510718

3’UTR 1

miRNA	3'UTR-1	MS2 control	enrichment 3'-1/MS2
hsa-miR-148b-3p	69611,22736	184,633848	377,0231088
hsa-miR-140-5p	26822,2468	207,713079	129,1312369
hsa-miR-15b-5p	1945,862377	23,079231	84,31227093
hsa-miR-15a-5p	3231,997441	46,158462	70,01960856
hsa-miR-16-5p	28674,81441	415,426158	69,02505742
hsa-miR-425-5p	6937,132652	115,396155	60,11580413
hsa-miR-30b-5p	1312,790713	23,079231	56,88190881
hsa-miR-148a-3p	348202,7429	10385,65395	33,52728143
hsa-miR-320d	559,768629	23,079231	24,25421492
hsa-miR-574-3p	2125,788008	92,316924	23,02706715
hsa-miR-29b-3p	1759,272834	92,316924	19,05688315
hsa-miR-339-5p	819,6612067	46,158462	17,75755021
hsa-miR-509-5p	406,4986472	23,079231	17,61317989
hsa-miR-181b-5p	6210,766217	369,267696	16,81914309
hsa-miR-9-5p	3485,226107	207,713079	16,77904022
hsa-miR-671-3p	766,3499087	46,158462	16,6025876
hsa-miR-107	346,523437	23,079231	15,014514
hsa-miR-320b	9669,336674	692,37693	13,96542296
hsa-miR-25-3p	6217,430129	623,139237	9,977593706
hsa-miR-103a-3p	11675,17426	1177,040781	9,919090697
hsa-miR-34a-5p	226,5730165	23,079231	9,817182231
hsa-miR-29a-3p	23710,19978	2492,556948	9,51240043
hsa-miR-103b	10688,91525	1153,96155	9,262800176
hsa-miR-362-5p	633,0716637	69,237693	9,143454039
hsa-miR-320a	36551,55869	4085,023887	8,947697663
hsa-miR-320c	1572,683291	184,633848	8,517849289
hsa-miR-509-3p	533,11298	69,237693	7,69975077
hsa-miR-506-3p	699,7107862	92,316924	7,579442164
hsa-miR-452-5p	173,2617185	23,079231	7,507257
hsa-miR-629-5p	2419,000147	346,188465	6,987523823
hsa-miR-125b-2-3p	159,933894	23,079231	6,929775693
hsa-miR-9-3p	159,933894	23,079231	6,929775693
hsa-miR-24-3p	17179,56578	3208,013109	5,355204357
hsa-miR-140-3p	2099,132359	392,346927	5,350194469
hsa-miR-92a-3p	27515,29368	5146,668513	5,3462339
hsa-miR-574-5p	366,5151737	69,237693	5,293578654
hsa-miR-3074-5p	16853,03408	3184,933878	5,291486331
hsa-miR-92b-3p	486,4655942	92,316924	5,269516933
hsa-miR-532-3p	1186,17638	230,79231	5,139583639
hsa-miR-23b-3p	353,1873492	69,237693	5,101084885
hsa-miR-181a-5p	1039,570311	207,713079	5,004838
hsa-miR-199b-5p	113,2865082	23,079231	4,908591116
hsa-miR-152-3p	779,6777332	161,554617	4,826093786
hsa-miR-509-3-5p	326,5317002	69,237693	4,716097346
hsa-miR-361-5p	106,622596	23,079231	4,619850462
hsa-miR-148a-5p	952,9394517	207,713079	4,587768167
hsa-miR-27b-5p	419,8264717	92,316924	4,547665298
hsa-miR-135b-5p	832,9890312	184,633848	4,511572717
hsa-miR-25-5p	413,1625595	92,316924	4,475480135
hsa-miR-501-5p	413,1625595	92,316924	4,475480135
hsa-miR-27b-3p	7223,680879	1800,180018	4,012754728
hsa-miR-32-5p	266,55649	69,237693	3,849875385
hsa-miR-30c-5p	5890,898429	1546,308477	3,809652806
hsa-miR-200c-3p	86,63085924	23,079231	3,7536285
hsa-miR-182-5p	506,457331	138,475386	3,657381616
hsa-miR-151a-3p	5977,529288	1707,863094	3,500004953
hsa-miR-30a-3p	159,933894	46,158462	3,464887846
hsa-miR-454-3p	146,6060695	46,158462	3,176147192
hsa-miR-671-5p	73,30303475	23,079231	3,176147192
novel_16	859,6446802	276,950772	3,103962029
hsa-miR-26b-5p	346,523437	115,396155	3,0029028
hsa-miR-4521	2199,091042	738,535392	2,977637993
hsa-miR-106b-3p	1572,683291	530,822313	2,962730187
hsa-miR-576-3p	199,9173675	69,237693	2,887406539
hsa-miR-130a-3p	66,6391225	23,079231	2,887406539
hsa-miR-29b-1-5p	66,6391225	23,079231	2,887406539
hsa-miR-506-5p	66,6391225	23,079231	2,887406539
hsa-miR-23a-3p	1126,20117	392,346927	2,870421794
hsa-miR-340-5p	1059,562048	369,267696	2,869360248
hsa-miR-584-5p	326,5317002	115,396155	2,829658408
hsa-miR-221-5p	579,7603657	207,713079	2,791159654
hsa-miR-21-3p	126,6143327	46,158462	2,743036212
hsa-miR-1180-3p	59,97521025	23,079231	2,598665885
hsa-miR-421	59,97521025	23,079231	2,598665885
hsa-miR-424-5p	119,9504205	46,158462	2,598665885
hsa-miR-186-5p	1925,87064	784,693854	2,454295558
hsa-miR-10a-5p	1652,650238	692,37693	2,386922739
hsa-miR-33a-3p	53,311298	23,079231	2,309925231
hsa-miR-33b-3p	53,311298	23,079231	2,309925231
hsa-miR-365a-3p	859,6446802	392,346927	2,19103202
hsa-miR-512-3p	99,95868374	46,158462	2,165554904
hsa-miR-27a-3p	5204,515467	2423,319255	2,147680483
hsa-miR-941	246,5647532	115,396155	2,136680839
hsa-miR-27a-5p	2179,099306	1061,644626	2,052569431
hsa-miR-185-5p	1319,454625	646,218468	2,041808909
hsa-miR-1255a	46,64738575	23,079231	2,021184577
hsa-miR-503-5p	46,64738575	23,079231	2,021184577
hsa-miR-183-5p	139,9421572	69,237693	2,021184577
hsa-miR-195-5p	619,7438392	0	n.d.
hsa-miR-31-5p	906,2920659	0	n.d.
hsa-miR-324-5p	1825,911956	0	n.d.
hsa-miR-328-3p	852,9807679	0	n.d.
hsa-miR-508-3p	3638,496088	0	n.d.
hsa-miR-522-3p	2598,925777	0	n.d.
