# Supplementary material for: Identification and characterization of novel CD274 (PD‐L1) regulating microRNAs and their functional relevance in melanoma
Source: Clin Transl Med. 2022 Jul 8;12(7):e934. doi: 10.1002/ctm2.934 (PMC9270002; doi:10.1002/ctm2.934)
Supplement: Supplementary file 9 — Supporting Information Table 1 – List of the primers used for cDNA synthesis and miRNA expression analysis through qPCR Supporting Information Table 2 – Selected list of the miRNAs found to target CD274 CDS and 3′‐UTR through RNAseq analysis after miTRAP. The absolute counts of the miRNA enrichment in the targeted (CDS or 3′‐UTR) and the control MS2 loop sequence are presented along with the fold enrichment of the miRNAs (ratio of enrichment in target sequence/enrichment in MS2 loop sequence) and the mature sequences of the miRNAs in question. Only the miRNAs that fit our selection criteria are shown. Supporting Information Table 3 – Cumulative list of the sequencing reads (target sequence and MS2 sequence) of all the miRNAs detected through RNAseq after miTRAP along with their fold enrichment. [file CTM2-12-e934-s007.docx]

Supplementary Table 1 – List of the primers used for cDNA synthesis and miRNA expression analysis through qPCR

Supplementary Table 2 – Selected list of the miRNAs found to target CD274 CDS and 3’-UTR through RNAseq analysis after miTRAP. The absolute counts of the miRNA enrichment in the targeted (CDS or 3’-UTR) and the control MS2 loop sequence are presented along with the fold enrichment of the miRNAs (ratio of enrichment in target sequence/enrichment in MS2 loop sequence) and the mature sequences of the miRNAs in question. Only the miRNAs that fit our selection criteria are shown.

Supplementary Table 3 – Cumulative list of the sequencing reads (target sequence and MS2 sequence) of all the miRNAs detected through RNAseq after miTRAP along with their fold enrichment.
